# A quest for greater thermodynamic rigour in the quantitative characterization of protein self-association by direct assessment of sedimentation equilibrium distributions

**DOI:** 10.1007/s12551-025-01371-1

**Published:** 2025-12-23

**Authors:** Donald J. Winzor, Peter R. Wills

**Affiliations:** 1 School of Chemistry and Molecular Biosciences, University of Queensland, Brisbane, Queensland , 4072 Australia; 2 Department of Physics, University of Auckland, Private Bag 92019, Auckland, New Zealand

**Keywords:** Protein self-association, Quantitative characterization, Sedimentation equilibrium, Statistical mechanics, Thermodynamic nonideality, Ultracentrifugation

## Abstract

This review summarizes research designed to enhance thermodynamic rigour in the quantitative characterization of protein self-association by direct analysis of sedimentation equilibrium distributions. The effects of thermodynamic nonideality have been incorporated into analytical approaches that not only afford experimental delineation of the monomer thermodynamic activity throughout a sedimentation equilibrium distribution but also take into account the composition dependence of species activity coefficients that arises from their consideration on the statistical-mechanical basis of excluded volume. Allowance for thermodynamic nonideality in terms of nearest-neighbor interactions should suffice for that iterative procedure. Attempts to eliminate the need for iterative analysis by expressing total protein concentration as a virial expansion in monomer activity have met with only limited success. The relevance of these scientific developments to their incorporation into the currently used numerical simulation approaches for the characterization of protein self-association by sedimentation equilibrium is also discussed.

## Introduction

Early analyses of sedimentation equilibrium distributions were based on the concept of a net balance between opposing centrifugal and diffusional steady state hydrodynamic fluxes (Lamm 1929). Indeed, the fact that both fluxes are always zero through the air–liquid meniscus and the cell base led to development of the approach-to-equilibrium method (Archibald 1947) for the quantitative characterization of reversible protein self-association (Klainer and Kegeles 1955, 1956). Interest in the use of the Archibald procedure waned with the realization (Goldberg 1953) that the analysis of sedimentation equilibrium distributions could be accorded full thermodynamic status by viewing the gradient in solute chemical potential as a diffusional force that balanced the “gravitational” and buoyant forces induced by the centrifugal field.

The next major theoretical breakthrough was the publication of a quantitative expression for direct analysis of the radial concentration dependence associated with attainment of sedimentation equilibrium (Haschemeyer and Bowers 1970)—a development that was soon followed by the report of an analytical procedure for evaluating the thermodynamic activity of monomer throughout the sedimentation equilibrium distribution for a self-associating protein (Milthorpe et al. 1975). Our aim since then has been to examine the extent to which greater thermodynamic rigour can be incorporated into the analysis of sedimentation equilibrium distributions reflecting protein self-association by incorporating the effect of thermodynamic nonideality on the statistical-mechanical basis of excluded volume. Advances in computer technology have concurrently revolutionized ultracentrifugal practice by virtue of the consequent ability to replace the need for analytical solutions by resorting to the numerical simulation procedures such as NONLIN (Johnson et al. 1981), SEDANAL (Sherwood and Stafford 2016a,b), SEDFIT (Schuck 2016), and SEDNTERP (Philo 2023). However, we have continued with the analytical approach for the current endeavour in order to retain model independence of the analysis.

This investigation also explores the possibility of further extending the analytical approach by taking advantage of its ability to delineate the concentration distribution for the smallest species from that reflecting the total concentration of a mixture of species. As noted in earlier studies of heterogeneous associations (Nichol et al. 1976; Wilson et al. 1997), subtraction of the concentration distribution for the smallest species from the total concentration yields a residual distribution from which the corresponding concentration of second-smallest species can be determined. Successive application of such a stepwise approach has potential for model-independent identification of the species involved in the self-associating system. A critical test of that potential is provided by examining the theoretical feasibility of distinguishing between two isodesmic self-association models (Jeffrey et al. 1976; Nichol et al. 1984) that have been proposed to account for the sedimentation equilibrium behaviour of zinc-free insulin at neutral pH.

## Theoretical aspects

### Expression for direct analysis of sedimentation equilibrium distributions

As first noted by Haschemeyer and Bowers (1970), the sedimentation distribution for a single nonassociating solute with molar mass ${M}_{i}$ and partial specific volume ${\overline{v} }_{i}$ that is subjected to centrifugation at angular velocity *ω* and absolute temperature *T* is described by the relationship

1 $${z}_{i}\left(r\right) ={z}_{i}({r}_{F}){\mathrm{exp}}\left[\frac{{M}_{i}(1 - {\overline{v} }_{i}{\rho }_{s}){\omega }^{2}({r}^{2} - {r}_{F}^{2})}{2RT}\right]$$

which describes the thermodynamic activity of solute *i* at radial position *r*, ${z}_{i}\left(r\right)$, in terms of the value, ${z}_{i}({r}_{F})$, at a selected reference radial distance ${r}_{F}$. ${\rho }_{s}$ is the solvent density (Wills and Winzor 1992; Wills et al. 1993) and *R* the universal gas constant. The thermodynamic activity is defined under the thermodynamic constraints of constant temperature, *T*, and chemical potential of solvent, *µ*_*s*_ (Wills and Winzor 1992; Wills et al. 1993). This thermodynamic activity is a molar parameter and is therefore most simply described as ${\gamma }_{i}{C}_{i},$ the product of a molar concentration (${C}_{i}$), and the corresponding dimensionless molar activity coefficient (${\gamma }_{i})$. After making the substitution (Wills et al. 1996)

2 $${\psi }_{i}\left(r\right)= {\mathrm{exp}}\left[\frac{{M}_{i}(1 - {\overline{v} }_{i}{\rho }_{s}){\omega }^{2}({r}^{2} - {r}_{F}^{2})}{2RT}\right]$$

and expressing molar concentration on the weight-concentration scale as ${c}_{i}/{M}_{i}$, Eq. (1) becomes

3 $$\frac{{c}_{i}(r)}{{M}_{i}}=\frac{{z}_{i}\left({r}_{F}\right){\psi }_{i}(r)}{{\gamma }_{i}(r)}$$

as the description of the concentration distribution for a single solute species.

For a solute undergoing reversible self-association, a corresponding relationship for $c_i(r)$ applies to each oligomeric state, but with the additional proviso that those $z_i(r)$ at any given radial distance may be expressed in terms of the association constant $K_i$ and the monomer activity raised to the appropriate power, $K_i{\lbrack z_1(r)\rbrack}^i$, to comply with the law of mass action. The expression analogous to Eq. (3) for total weight-concentration of solute, $\overline c(r)$, is then given by 

4a $$\frac{\overline{c }(r)}{{M}_{1}} = \frac{{z}_{1}\left({r}_{F}\right){\psi }_{1}( r)}{{\gamma }_{1}(r)} + \frac{{2K}_{2}[{z}_{1}\left({r}_{F}\right){\psi }_{1}( r){]}^{2}}{{\gamma }_{2}(r)} +\frac{3{K}_{3}[{z}_{1}({r}_{F}){\psi }_{1}(r){]}^{3}}{{\gamma }_{3}(r)} +\dots$$

which clearly requires the assignment of magnitudes to the various activity coefficients at each radial position as a prerequisite for quantitative characterization of the protein self-association. To conform with the customary experimental practice of employing weight concentrations, the thermodynamic activity is also expressed on the same basis (g/L), whereupon Eq. (4a) can also be written in the form

4b $$\overline c\left(r\right)=\frac{M_1z_1\left(r\right)\psi_1(r)}{\gamma_1(r)}+\frac{X_2\lbrack\lbrack M_1z_1(r_F)\psi_1(r)\rbrack^2}{\gamma_2(r)}+\frac{X_3\left[M_1z_1\left(r_F\right)\psi_1\right)r)]^3}{\gamma_3}+...$$

with the association constant ${X}_{i}=i{K}_{i}/{M}_{1}^{i-1}$ also expressed on the weight-concentration basis, that is, in units of ${\mathrm{L}}^{i-1}{\mathrm{g}}^{1-i}$.

### Allowance for thermodynamic nonideality

In the earliest allowances for thermodynamic nonideality effects (Adams and Fujita 1963), an assumption was made that the activity coefficients of oligomers could be related by the expression

5 $${\gamma }_{i}\left(r\right)=\mathrm{exp}[iB{M}_{1}\overline{c }\left(r\right)]$$

where *B* was an empirical constant to be evaluated as an additional curve-fitting parameter. This approach was widely used to adjust the magnitude of the apparent association constant (ratio of concentrations raised to the appropriate power): indeed, it is incorporated into the NONLIN (Johnson et al. 1981), ORIGIN (McRorie and Voelker 1993), and INVEQ (Rowe 2005) software as an addition to the already large number of curve-fitting parameters to be deduced from the analysis. In retrospect, the survival of the Adams-Fujita relationship for three decades was surprising in that any change in the returned association constant merely demonstrated nonconformity of the experimental system with the assumption inherent in Eq. (5) that the activity coefficient ratio, ${\gamma }_{i}(r)/[{\gamma }_{1}(r){]}^{i}$, is unity, thereby rendering the measured equilibrium constant insensitive to effects of thermodynamic nonideality (Jacobsen and Winzor 1992).

A better approach employs expressions emanating from consideration of thermodynamic nonideality on the statistical-mechanical basis of the potential-of-mean-force between molecules (McMillan and Mayer 1945; Hill 1968; Wills and Winzor 2005). In this excluded volume theory, the activity coefficient of a chemically inert species *i* at radial distance *r* is defined by the relationship (Hill 1959) 

6 $${\gamma }_{i}(r)={\mathrm{exp}} \left[\frac{2{B}_{ii}^{*}{c}_{i}(r)}{{M}_{i}}+\sum_{j\ne i}\frac{{B}_{ij}^{*}{c}_{i}{\left(r\right)c}_{j}(r)}{{M}_{i}{M}_{j}}+ \dots \right]$$

in which the osmotic second virial coefficients ${B}_{ii}^{*}$ and ${B}_{ij}^{*}$ stem from the expression of osmotic pressure as a power series in concentration: specifically,

7 $$\frac{\Pi }{RT}=\sum_{i}\frac{{c}_{i}}{{M}_{i}}+\sum_{i}{B}_{ii}^{*}{\left(\frac{{c}_{i}}{{M}_{i}}\right)}^{2}+\sum_{j\ne i}{B}_{ij}^{*}\left(\frac{{c}_{i}{c}_{j}}{{M}_{i}{M}_{j}}\right)+\dots$$

They find simple statistical-mechanical interpretation in terms of the physical interactions between pairs (*ij*) of solute molecules (McMillan and Mayer 1945; Hill 1959). For two spherical molecules with radii ${R}_{i}$ and ${R}_{j}$, the osmotic second virial coefficient for mutual interaction ${B}_{ij}^{*}$ is given (McMillan and Mayer 1945) by

8a $${B}_{ij}^{*}=\left(\frac{2}{3}\right)\left(2-{\delta }_{ij}\right)\pi {N}_{A}({R}_{i}+{R}_{j}{)}^{3}-2(2-{\delta }_{ij})\pi {N}_{A} \underset{({R}_{i}+{R}_{j})}{\overset{\infty }{\int }}{f}_{ij}(x){x}^{2}dx$$

where ${\delta }_{ij}\text{ is the Kronecker function}$ (equal to unity for *i* = *j*, otherwise zero). The Mayer f-function ${f}_{ij}(x)$ is defined as

8b $${f}_{ij}\left(x\right)=\mathrm{exp}\left[-\frac{{u}_{ij}\left(x\right)}{{k}_{B}T}\right]-1$$

in which ${u}_{ij}(x)$ specifies the potential energy of the two molecules as a function of the centre-to-centre separation $x$, and ${k}_{B}$ is the Boltzmann constant. Avogadro’s number ${N}_{A}$ is included in Eq. (8a) to convert the second virial coefficient from a molecular to a molar basis.

For charged spherical molecules, the energy function ${u}_{ij}(x)$ can be described in terms of the Debye-Hückel inverse-screening length *κ*, the dielectric constant of the solvent *ε*, electronic charge *e*, and the net charges ${Z}_{i}$, ${Z}_{j}$ of the molecules by

8c $${u}_{ij}\left(x\right)=\frac{{Z}_{i}{Z}_{j}{e}^{2}\mathrm{exp}[-\kappa \left(x-{R}_{i}-{R}_{j}\right)]}{\varepsilon \left(1+\kappa {R}_{i}\right)\left(1+\kappa {R}_{j}\right)x};\;x \ge {R}_{i}+{R}_{j}$$

On taking advantage of the relationship between the molar ionic strength ${I}_{M}$ and the inverse screening length,

9a $${\kappa }^{2}=\frac{8\pi {N}_{A}{e}^{2}{I}_{M}}{\varepsilon {k}_{B}T}$$

we obtain the expression

9b $$\frac{{u}_{ij}(x)}{{k}_{B}T}=\frac{1000{Z}_{i}{Z}_{j}{\kappa }^{2}\mathrm{exp}[-\kappa \left(x-{R}_{i}-{R}_{j}\right)]}{8\pi {N}_{A}{I}_{M}\left(1+\kappa {R}_{i}\right)\left(1+\kappa {R}_{j}\right)x}$$

in which the magnitude (in cm^−1^) of the inverse screening length *κ* (Eq. (9a)) is calculated from the molar ionic strength ${I}_{M}$ as $3.27\times {10}^{7}\sqrt{{I}_{M}}$ for *T* = 20 °C: the factor 1000 in the numerator of the third term arises from the molar definition of *I*_*M*_, and the units of cm^−3^ for ${\kappa }^{3}$ (Wills and Winzor 2009). An approximate analytical expression for evaluating ${B}_{ij}^{*}$ is obtained by expanding the exponential in Eq. (8b) as

10 $$f_{ii}\left(x\right)=-\frac{u_{ii}(x)}{k_BT}+\frac12\left[\frac{u_{ii}(x)}{k_BT}\right]^2-...$$

whereupon the expression for the second virial coefficient deduced from Eq. (8a) becomes (Wills and Winzor 2009; Scott et al. 2010)

11 $${B}_{ii}^{*}=\frac{16\pi {N}_{A}{R}_{i}^{3}}{3} + \frac{{Z}_{i}^{2}(1+2\kappa {R}_{i})}{4{I}_{M}(1+\kappa {R}_{i}{)}^{2}} - \frac{{Z}_{i}^{4}(1000{\kappa }^{3})}{128\pi {N}_ {A}{I}_{M}^{2}(1+\kappa {R}_{i}{)}^{4}} +\dots$$

The corresponding approximate expression for the second virial coefficient for physical interaction between two different species is (Wills and Winzor 2009; Scott et al. 2010)

12 $${B}_{ij}^{*}= \frac{4\pi {N}_{A}({R}_{i}+{R}_{j}{)}^{3}}{3} + \frac{{Z}_{i}{Z}_{j}(1+\kappa {R}_{i}+\kappa {R}_{j})}{2{I}_{M}\left(1+\kappa {R}_{i}\right)(1+\kappa {R}_{j})} - \frac{{Z}_{i}^{2}{Z}_{j}^{2}(1000{\kappa }^{3})}{64\pi {N}_{A}{I}_{M}^{2}(1+\kappa {R}_{i}{)}^{2}(1+\kappa {R}_{j}{)}^{2}} +\dots$$

where ${R}_{j}$ and ${Z}_{j}$ denote the respective radius and charge of the second species. For proteins with a reasonably uniform charge distribution, the activity coefficients in Eq. (6) for monomer and polymer (n-mer) can be obtained with an acceptable degree of precision by combining measurements of the Stokes radius and net charge of monomer with the concepts of spherical geometry, ${R}_{n}={n}^{1/3}{R}_{1}$, and charge conservation, ${Z}_{n}=n{Z}_{1}$.

### Analytical characterization of protein self-association

A breakthrough in direct analysis of sedimentation equilibrium distributions for the characterization of protein self-association was the development of a procedure for the experimental determination of ${M}_{1}{z}_{1}({r}_{F})$, the thermodynamic activity of monomer, as a proportion of the total protein concentration $\overline{c }({r}_{F})$ at a selected radial distance ${r}_{F}$. However, the significance of that Omega analysis (Milthorpe et al. 1975; Wills et al. 1980) was not realized by the ultracentrifugal research community. Indeed, a footnote in a review of sedimentation equilibrium (Minton 1990) noted that use of the procedure appeared to be geographically restricted.

The Omega function is an experimental parameter defined by the relationship

13 $$\Omega \left(r\right)=[\overline{c }(r)/\overline{c }({r}_{F})]{\mathrm{exp}} \left[\frac{{M}_{1}\left(1-\overline{v}{\rho }_{s}\right){\omega }^{2}({r}_{F}^{2}-{r}^{2})}{2RT}\right]$$

which, on the basis of Eq. (2), may also be written as

14 $$\Omega (r)=\frac{\overline{c }(r)/\overline{c }({r}_{F})}{{\psi }_{1}(r)}$$

On the grounds that $\overline{c }(r)$ → ${z}_{1}$(*r*) in the limit of zero solute concentration, it can then be argued that the ordinate intercept of the dependence of $\Omega \left(r\right)$ upon $\overline{c }(r)$, ${\Omega }_{o}$ becomes

15 $$\Omega_o=\frac{M_1z_1(r)/\overline c(r_F)}{\psi_1(r)}={M_1z}_1(r_F)/\overline c(r_F)$$

where the alternative form is obtained by the substitution of Eq. (1) with *i* = 1 for ${z}_{1}\left(r\right).$ Combination of ${z}_{1}\left({r}_{F}\right)$ as ${\Omega }_{o}\overline{c }({r}_{F})$ with Eq. (1) then leads to delineation of ${M}_{1}{z}_{1}(r)$ throughout the sedimentation equilibrium distribution, and hence to characterization of the self-association by an iterative process. For a system with total protein concentration described by Eq. (4b), the corresponding expression in terms of the Omega function becomes

16 $$\Omega \left(r\right)={\Omega }_{o}+{X}_{2}^{app}{\Omega }_{o}^{2} [\overline{c }({r}_{F}){\psi }_{1}(r){]}^{2}+{X}_{3}^{app}{\Omega }_{o}^{3}[\overline{c }({r}_{F}){\psi }_{1}\left(r\right){]}^{3}+\dots$$

where ${X}_{i}^{app}={X}_{i}[\{{\gamma }_{1}\left(r\right){\}}^{i}/{\gamma }_{i}(r)]$ is the apparent association constant for the reaction $iA {\leftrightarrow A}_{i}$ expressed on the weight concentration scale. The thermodynamic association constants are then obtained by iterative, nonlinear curve-fitting in terms of Eqs. (7), (11), (12), and (16) (Wills et al. 1980). An obvious advantage of this analytical approach over the more popular numerical integration procedures is its ability to provide direct experimental determination of the $z_1\left(r\right)\; vs\; \overline c(r)$ relationship.

A drawback of this Omega analysis is the extent of reliance placed upon the accuracy of the extrapolation to obtain ${\Omega }_{o}$ as the ordinate intercept of the dependence of $\Omega (r)$ upon $\overline{c }(r)$ (Morris and Ralston 1985, 1989). However, that difficulty can be overcome (Jacobsen and Winzor 1992) by obtaining ${\Omega }_{o}$ as the ordinate intercept of the dependence of $\Omega \left(r\right)$ upon $\overline{c }\left({r}_{F}\right){\psi }_{1}\left(r\right)$, which is linear for ideal two-state self-association and rendered only slightly curvilinear by incorporating the consequences of thermodynamic nonideality.

### Self-association as a form of thermodynamic nonideality

An obvious disadvantage of this statistical-mechanical approach to allow for effects of thermodynamic nonideality is expression of the activity coefficient for a species in terms of the concentrations of all species present. By regarding solute self-association as another form of thermodynamic nonideaality, Hill and Chen (1973) proposed an alternative statistical-mechanical approach in which the activity coefficient and hence thermodynamic activity of monomer were expressed in terms of total concentration of the single self-associating solute. The resultant expression for the activity of monomer in terms of base-molar solute concentration,$\overline{C }=\overline{c }/{M}_{1}$, is

17 $${z}_{1}(r)= \overline{C }(r)\mathrm{exp}\left[2{B}_{2}\overline{C }\left(r\right)+\frac{3}{2}{B}_{3}[\overline{C }\left(r\right){]}^{2}+\dots \right]$$

where

18a $${B}_{2}=-{K}_{2}+{B}_{11}^{*}$$

18b $${B}_{3}=-2{K}_{3}+4{K}_{2}^{2}-2{K}_{2}\left(4{B}_{11}^{*}-{B}_{12}^{*}\right)+{B}_{111}^{*}$$

As in Eq. (4a), ${K}_{2}$ and ${K}_{3}$ are the respective molar thermodynamic association constants for the formation of dimer and trimer from monomer, while ${B}_{11}^{*}$ and ${B}_{12}^{*}$ remain the osmotic second virial coefficients for physical monomer − monomer and monomer − dimer interactions defined in Eq. (8a). Its counterpart for ${B}_{111}^{*}$, the osmotic third coefficient for trimer, is (Hill 1956)

19 $${B}_{111}^{*}=-\frac{8}{3}{\pi }^{2}{N}_{A}^{2}{\int }_{0}^{\infty }{x}_{ij}f\left({x}_{ij}\right)d{x}_{i}{\int }_{0}^{\infty }{x}_{jk}f\left({x}_{jk}\right)d{x}_{jk}{\int }_{\mid {x}_{ij}-{x}_{jk}\mid }^{{x}_{ij}+{x}_{jk}}{x}_{ik}f\left({x}_{ik}\right)d{x}_{ik}$$

which reflects the interactions between pairs in a cluster of three monomer molecules (*i*, *j*, and *k*), This third virial coefficient must always be evaluated by numerical integration whenever molecular interactions forces other than hard-sphere excluded volume come into play. Despite its obvious relevance to the allowance for effects of thermodynamic nonideality in sedimentation equilibrium distributions, this suggested procedure only resurfaced two decades later in investigations (Wills and Winzor 1992; Wills et al. 1996) that provided a more detailed account of its derivation from the statistical-mechanical approach already discussed.

The starting point is the statement of mass conservation for nonideal self-association of a protein present at base-molar concentration $\overline{C }=\overline{c }/{M}_{1}$, namely, 

20 $$\overline{C }=\frac{{z}_{1}}{{\gamma }_{1}}+\frac{2{z}_{2}}{{\gamma }_{2}}+\frac{3{z}_{3}}{{\gamma }_{3}}+...={z}_{1}\left[\frac{1}{{\gamma }_{1}}+\frac{2{K}_{2}{z}_{1}}{{\gamma }_{2}}+\frac{3{K}_{3}{z}_{1}^{2}}{{\gamma }_{3}}+...\right]$$

where molar concentrations of species ${C}_{i}$ (*i* = 1, monomer; *i* = 2, dimer, etc.) are expressed as the ratio of their thermodynamic activities ${z}_{i}$ and their activity coefficients ${\gamma }_{i}$. Incorporation of the expressions for activity coefficients (Eq. (6)) into Eq. (20) leads to the truncated relationship. 

21 $$\frac{{z}_{1}(r)}{\overline{C }(r)}={\mathrm{exp}}\left[\begin{array}{c}2\left({B}_{11}^{*}-{K}_{2}\right){C}_{1}\left(r\right)+\\ \left\{\left(\frac{3}{2}\right){B}_{111}^{*}-8{K}_{2}{B}_{11}^{*}+3{K}_{2}{B}_{12}^{*}+2{K}_{2}^{2}+3{K}_{3}\right\}[{C}_{1}(r){]}^{2}+ \dots \end{array}\right]$$

Conversion of this power series in ${C}_{1}(r)$ to the required one in $\overline{C }(r)$ is effected by taking into account the extra terms in ${C}_{2}$ that are introduced, the result being

22 $$\frac{{z}_{1}(r)}{\overline{C }(r)}=\mathrm{exp}\left[\begin{array}{c}2({B}_{11}^{*}-{K}_{2}) \overline{C }\left(r\right) +\left(\frac{3}{2}\right)\{4{K}_{2}^{2} -2{K}_{2}(4{B}_{11}^{*}-{B}_{12}^{*}) \\ + {B}_{111}^{*}-2{K}_{3}\}[\overline{C }(r){]}^{2} + \dots \end{array}\right]$$

which is the Hill − Chen expression for the dependence of the thermodynamic activity of monomer as a function of the base-molar protein concentration.

A problem with this approach is its reliance on rapid convergence of the virial expansion series—a requirement that is only met for relatively weak self-associations (Wills and Winzor 1992; Wills et al. 1996). Fortunately, that limitation can be overcome to some extent by reframing the Hill − Chen approach in terms of an expression for $\overline{C }$ as a polynomial function of ${z}_{1}.$ The starting point for this treatment is the relationship (*cf *Eq. (17)).

23a $$\overline{C }={z}_{1}(1+2{b}_{2}{z}_{1}+3{b}_{3}{z}_{1}^{2}+\dots$$

where the coefficients ${b}_{2} \;\mathrm{and}\; {b}_{3}$ come from the virial expansion of osmotic pressure in terms of monomer activity,

23b $$\frac{\Pi }{RT}={z}_{1}+{b}_{2}{z}_{1}^{2}+{b}_{3}{z}_{1}^{3}+...$$

and the relationship (Hill 1959)

23c $$\overline{C }={z}_{1}\partial \left(\frac{\Pi }{RT}\right)/\partial {z}_{1}$$

These coefficients are related to the traditional osmotic virial coefficients (Eqs. (8a and (19)) for a self-associating system by the expressions

23d $${b}_{2}=-{B}_{2}={K}_{2}-{B}_{11}^{*}$$

23e $${b}_{3}=-\frac{{B}_{3}}{2}+2{B}_{2}^{2}={K}_{3}-{K}_{2}{B}_{12}^{*}+2({B}_{11}^{*}{)}^{2}-\frac{{B}_{111}^{*}}{2}$$

In terms of the analysis of sedimentation equilibrium distributions, the counterpart of Eq. (4a) for a self-associating system then becomes

24 $$\overline C(r)=z_1{(r}_F)\psi_1(r)+2b_2\left[z_1{(r}_F\right)\psi_1(r)\rbrack^2+3b_3\left[z_1{(r}_F\right)\psi_1(r)\rbrack^3+...$$

which provides a means of determining the monomer thermodynamic activity at the reference radial position, ${z}_{1}{(r}_{F})$, as the ordinate intercept of the dependence of $\overline{C }(r)/{\psi }_{1}(r)$ upon ${\psi }_{1}(r)$. Global analysis of different sedimentation equilibrium distributions is effected by selecting the reference radial position on the basis of a common total protein concentration $\overline{c }(r)$.

### Experimental aspects

The currently recommended experimental procedure for characterizing self-association entails the recording of equilibrium distributions for samples with a range of protein concentrations subjected to centrifugation at multiple rotor speeds. This protocol was developed by Johnson et al. (1981) as a general procedure that eliminated the need for information on monomer size or stoichiometry of self-association. A different stance had been adopted in the alternative procedure for self-association characterization by direct analysis of equilibrium concentration distributions (Milthorpe et al. 1975; Wills et al. 1980, 1996), where the number of sedimentation equilibrium distributions was decreased markedly by using available information about the system to optimize the experimental design. Because the analytical approach is unfamiliar territory for most ultracentrifuge users, it is appropriate to precede any application of the above theory by reiterating some of those experimental considerations involved in optimizing the design of a sedimentation equilibrium run.

### Optimal design of sedimentation equilibrium experiments

The first consideration is the type of experiment required to provide the most reliable delineation of the $r\;vs\;\overline{c }(r)$ distribution for assessing the stoichiometry and strength of protein self-association. In that regard, the absorption optical system of the Beckman XL-I analytical ultracentrifuge records the radial dependence of absorbance, ${A}_{\lambda }\left(r\right)\;vs\;r$, that is readily converted to its concentration counterpart, $\overline{c }\left(r\right)\; vs\;r$, via the protein extinction extinction coefficient at wavelength *λ* for experiments of low speed (Van Holde and Baldwin 1958) and meniscus depletion (Yphantis 1964) design. Although equilibrium distributions recorded by the Rayleigh interference optical systems are usually employed because of their greater potential precision, they provide a measure of the difference between the protein concentration $\overline{c }\left(r\right)$ at radial distance *r* and that, $\overline{c }({r}_{a}$), at the air − liquid meniscus located at radial distance ${r}_{a}$. Consequently, the number of Rayleigh fringes observed, $j(r)$, ranges between zero at ${r}_{a}$ and $j({r}_{b})$ at the other extremity of the liquid column subjected to sedimentation equilibrium. From the viewpoint of defining the Rayleigh counterpart of the absolute protein concentration distribution, the required radial dependence is that of $J\left(r\right)=j\left(r\right)+J({r}_{a}$), where $J({r}_{a}$) is the protein concentration expressed in terms of Rayleigh fringes $[J({r}_{a})=3.33\overline{c }\left({r}_{a}\right)]$ at the air − liquid meniscus. Only in the event that a sufficiently high rotor speed is used to ensure a value of essentially zero for $J({r}_{a}$) may $j({r}_{a}$) be equated with $J({r}_{a}$) (Yphantis 1964). Use of low-speed equilibrium distributions (Van Holde and Baldwin 1958) is thus conditional upon the assignment of a magnitude to $J({r}_{a}$).

As recognized at the outset (Richards and Schachman 1959), the most accurate procedure for its experimental estimation entails the conduct of a synthetic boundary run on the solution subjected to sedimentation equilibrium in order to define the Rayleigh fringe equivalent $$({J}_{o})$$ of the loading concentration. This additional information allows $$J({r}_{a}$$) to be determined on the basis of mass conservation in that $${J}_{o}({r}_{b}^{2}-{r}_{a}^{2})$$ accounts for the total amount of solute, which is greater than the amount defined by the integral of $${r}^{2}dj$$ across the entire column by $$J({r}_{a}$$)($${r}_{b}^{2}-{r}_{a}^{2})$$. The Rayleigh fringe counterpart of the concentration at the air–liquid meniscus is then obtained from the expression

25 $$J\left({r}_{a}\right)={J}_{o}-\left[\frac{j({r}_{b}){r}_{b}^{2}-\underset{0}{\overset{j({r}_{b})}{\int }}{r}^{2}dj}{{r}_{b}^{2}-{r}_{a}^{2}}\right]$$

whereupon the absolute concentration (in Rayleigh fringe terms) becomes $J\left(r\right)=J({r}_{a}$) + $j\left(r\right).$ Alternatively, a more direct procedure for determining $J(r)$ entails location of the hinge point, the radial position ${r}_{H}$ at which the protein concentration remains invariant throughout the approach to sedimentation equilibrium, and hence the radial position at which $J\left(r\right)={J}_{o}$ (Richards and Schachman 1959). In a rare example of the application of this approach to sedimentation equilibrium distributions obtained with the Beckman XL-I instrument (Hall et al. 1999), the hinge point was obtained by overlaying an absorption distribution obtained in the initial stage of centrifugation upon its counterpart obtained at sedimentation equilibrium. In this approach, which also requires the synthetic boundary run to define ${J}_{o}$, the expression for absolute concentration (in fringes) is $J\left(r\right)={J}_{o}+j\left(r\right)-j({r}_{H})$. Need for the integration step in Eq. (25) is thereby avoided.

The above procedures for determining the meniscus concentration have essentially disappeared with the demise of the Beckman model E analytical ultracentrifuge in that the synthetic boundary experiment does not form part of the protocol on which the online software packages (NONLIN, SEDANAL, SEDFIT, SEDNTERP, etc.) are based. Instead, it is presumed that $J(r_a)$ for each distribution can be evaluated with sufficient accuracy as an additional parameter to emanate from nonlinear curve-fitting of the $\lbrack r,j(r)\rbrack$ distribution to the fringe counterpart of Eq. (4b), namely

26 $$j\left(r\right)+J\left(r_a\right)=J_1\left(r_F\right)(\psi_1\left(r\right)+X_2^\dagger\lbrack J_1\left(r_F\right)(\psi_1\left(r\right)\rbrack^2+...$$

where, for an ideal system, $$X_i^\dagger$$ = $${J}_{i}\left(r\right)/[{J}_{1}(r){]}^{i}$$ fringe^(*i*−1)^ is related to the corresponding association constant $${X}_{i}$$ [(L/g$${)}^{i-1}$$] for the formation of oligomer *i* from monomer by the expression $$X_i^\dagger$$=$${X}_{i}$$/[3.3$${3}^{(i-1)}]$$. To that end, an investigation (Hall et al. 1999) designed to test the validity of that presumption for a simpler, non-associating protein (ovalbumin) has revealed a 10% underestimation of $$J\left({r}_{a}\right)$$ by this means. Furthermore, removal of the inherent interdependence of molecular mass and $$J\left({r}_{a}\right)$$ estimates (Teller et al. 1969) by means of the MSTAR approach (Creeth and Harding 1982; Schuck et al. 2014) has shown that the situation is only improved in situations where $$J\left({r}_{a}\right)$$ is much smaller than $$j\left({r}_{b}\right)-j\left({r}_{a}\right)$$ or, in other words, where the sedimentation equilibrium distribution approaches that obtained by the meniscus depletion approach (Yphantis 1964). Where possible, the most logical approach for characterizing protein self-association equilibria by sedimentation equilibrium is therefore to restrict the choice of rotor speed to values yielding high-speed distributions in which the availability of a solvent baseline allows the identification of $$J\left(r\right)$$ with $$j\left(r\right)$$ because $$J\left({r}_{a}\right)$$ is effectively zero.

### Transition from Omega to Psi analysis

As mentioned above, the evaluation of monomer activity, ${z}_{1}\left(r\right)$, as a function of total concentration, $\overline{c }$(*r*), by means of the Omega function (Milthorpe et al. 1975) paved the way for direct analysis of sedimentation equilibrium concentration distributions for self-associating systems. The key to that procedure, described in Eqs. (13) − (16), is the determination of ${z}_{1}({r}_{F})$/$\overline{c }({r}_{F})$ as the ordinate intercept, ${\Omega }_{o}$, of the dependence of $\Omega \left(r\right)=[\overline{c }\left(r\right)/\overline{c }({r}_{F})]/{\psi }_{1}(r)$ upon total protein concentration $\overline{c }(r)$.

Inasmuch as $\overline{c }({r}_{F})$ is a constant, Eq. (14) may be arranged as 

27 $$\Omega \left(r\right)\overline{c }({r}_{F})=\frac{\overline{c }(r)}{{\psi }_{1}(r)}$$

in which the right-hand side is the ordinate parameter in the Psi procedure for the determination of ${z}_{1}({r}_{F}$) as the ordinate intercept of the dependence of $\overline{c }(r)/{\psi }_{1}(r)$ upon ${\psi }_{1}\left(r\right)$ (Wills et al. 1996). The essential difference between the two analyses of sedimentation equilibrium distributions thus resides in the use of $\overline{c }(r)$ as the abscissa in the Omega procedure—an action that has left the method devoid of an independent variable. That problem does not arise in the Psi procedure (the dependence of $\overline{c }(r)/{\psi }_{1}(r)$ upon ${\psi }_{1}(r)$) because ${\psi }_{1}(r)$ is an exact transformation of the independent variable (radial distance *r*) in an experiment on a solute with defined buoyant molecular mass that is conducted at fixed angular velocity *ω* and constant temperature *T* (Eq. (2)).

**Fig. 1:**
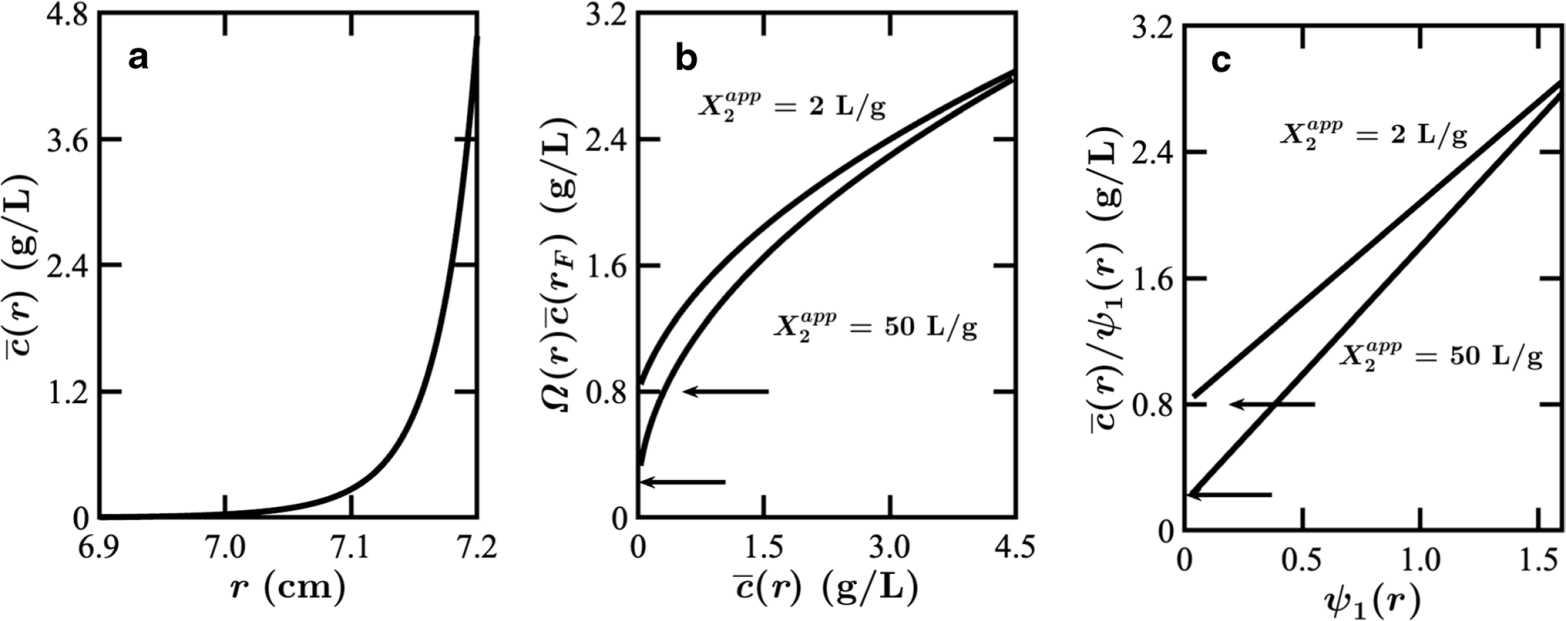
Comparison of the Omega and Psi analyses for the determination of monomer thermodynamic activity from sedimentation equilibrium distributions for a dimerizing protein. **a** Simulated concentration distribution for an ideal system with ${M}_{1}\left(1-\overline{v }\rho \right)=\mathrm{6,500}$ Da and ${X}_{2}^{app}=$ 2 L/g subjected to centrifugation at 30,000 rpm and 20 °C. **b** Omega analysis (with ${r}_{F}=$ 7.175 cm) of that distribution (upper curve) and its counterpart with ${X}_{2}^{app}$ raised to 50 L/g (lower curve): horizontal arrows denote the theoretical ordinate intercepts. **c** Corresponding Psi analyses of the same distributions

There is also an experimental reason for choosing the Psi approach in preference to the Omega method, where ever-increasing downward curvature of the *Ω*(*r*) $$vs\;\overline{c }(r)$$ dependence at low $$\overline{c }(r)$$ renders difficult the precise location of its ordinate intercept ($${\Omega }_{o}$$)—particularly in situations involving strong self-association (Morris and Ralston 1985, 1989). This experimental limitation of the Omega procedure is now illustrated by the application of both approaches to simulated sedimentation equilibrium distributions of meniscus-depletion design (Yphantis 1964), which allow more accurate definition of $$\overline{c }(r)$$ in an experimental context because of greater certainty about its value (zero) at the air − liquid meniscus in Rayleigh interference records of the distribution. The advantages of employing a high-speed sedimentation equilibrium distribution are evident from simulations based on an ideal system with the dimerization characteristics of α-chymotrypsin in acetate-chloride buffer, pH 3.9, *I*_*M*_ 0.2 (Tellam et al. 1979): $${M}_{1}(1-\overline{v}{\rho }_{s})$$ = 6500 Da, $${X}_{2}^{app}=$$$${c}_{2}/{c}_{1}^{2}$$ = 2 L/g. The pattern presented in Fig. 1a is the simulated sedimentation equilibrium distribution for a 3-mm column (6.9 ≤ *r* ≤ 7.2 cm) subjected to centrifugation at 20 °C and 30,000 rpm. An upper-limiting concentration of about 4.5 g/L was achieved by selecting a monomer concentration $${c}_{1}({r}_{F})$$ of 0.8 g/L with $${r}_{F}$$ = 7.175 cm as the reference radial position: the corresponding value of $$\overline{c }({r}_{F})$$ is 2.08 g/L.

Results from Omega analysis of the distribution are presented as the upper curve in Fig. 1b, where the product $\Omega \left(r\right)\overline{c }({r}_{F})$ has been used as the ordinate parameter to render its magnitude identical with that of $\overline{c }(r)/{\psi }_{1}(r)$, its counterpart in the Psi analysis (see Eq. (27)). Truncation of the plot at a value of 0.03 g/L for $\overline{c }(r)$, corresponding to 0.10 Rayleigh fringes, reflects the existence of an estimated random error of 0.02 fringes (0.006 g/L) in the delineation of sedimentation equilibrium distributions by the current Rayleigh interference optical system (Rowe 2005). Despite downward curvature of the plot, an estimate of ${{M}_{1}z}_{1}\left({r}_{F}\right)$, taken as ${c}_{1}({r}_{F})$, is very close to the input value of 0.8 g/L (horizontal arrow) that would emanate from extrapolation of the curve to deduce the ordinate intercept. Indeed, this procedure was used to deduce the reference monomer concentration for the α-chymotrypsin system on which the current simulations are based (Tellam et al 1979).

The corresponding estimate of ${c}_{1}({r}_{F})$ by Psi analysis as the ordinate intercept of the same [$r, \overline{c }(r)$] data set in terms of Eq. (4b) truncated at the dimer term (upper line in Fig. 1c) is rendered even more certain by the mandatory requirement of a linear dependence of $\overline{c }(r)/{\psi }_{1}(r)$ upon ${\psi }_{1}(r)$ with ordinate intercept ${c}_{1}({r}_{F})$ and slope ${c}_{2}({r}_{F})$. Use of the Psi approach has thus not only led to the evaluation of ${c}_{1}({r}_{F})$ but has also identified the self-association as a monomer − dimer equilibrium.

Although the Omega analysis has yielded a satisfactory estimate of ${c}_{1}({r}_{F})$ for this system with ${X}_{2}^{app}=2$ L/g, the required ordinate intercept becomes increasingly difficult to ascertain for systems with larger values of the dimerization constant. That difficulty is illustrated by increasing ${X}_{2}$ to 50 L/g and generating a simulated distribution very similar to that in Fig. 1a by lowering ${c}_{1}({r}_{F})$ to 0.18 g/L: $\overline{c }({r}_{F})$ then becomes 1.8 g/L for the same reference radial position (7.175 cm). For this stronger self-associating system, the likelihood of extrapolating the Omega plot (lower curve in Fig. 1b) to identify ${c}_{1}({r}_{F})$ as the mandatory ordinate intercept (signified by the horizontal arrow) is much decreased—even more so should the consequences of experimental uncertainty in $\overline{c }(r)$ be incorporated. On the other hand, the Psi analysis (lower line in Fig. 1c) continues to yield good estimates of ${c}_{1}({r}_{F})$ and ${c}_{2}({r}_{F})$, particularly when account is taken of the fact that the relatively larger consequences of experimental scatter can be eliminated by removing those poorly defined [${\psi }_{1}\left(r\right), \overline{c }(r)/{\psi }_{1}(r)]$ data from the set used for the determination of the ordinate intercept and slope by linear least-squares analysis. That option is not available in the Omega analysis, where the extrapolation to obtain ${c}_{1}({r}_{F})$ has to be based solely on the poorly defined [$\overline{c }\left(r\right), \Omega \left(r\right)\overline{c }(r)$] data at low $\overline{c }(r)$. In that regard, the transition from Omega to Psi for subsequent analyses of sedimentation equilibrium distributions for interacting systems has not heralded the demise of the Omega approach but rather its reincarnation in a better form.

### Applications of the analytical approach

In early applications of the analytical approach to the characterization of two-state protein self-association by direct analysis of sedimentation equilibrium distributions, the dependence of monomer thermodynamic nonideality, ${M}_{1}{z}_{1}\left(r\right),$ upon total protein concentration $\overline{c }(r)$ obtained by the Omega procedure was often identified with that of monomer concentration ${c}_{1}(r)$ to allow the determination of an apparent association constant ${X}_{i}^{app}$ on the basis of thermodynamic ideality (Tellam et al. 1978, 1979). A major exception was the investigation of lysozyme self association (pH 8.5, ${I}_{M}$ 0.15, 15 °C), in which initial estimates of ${X}_{2}^{app}$ and ${X}_{3}^{app}$ were obtained by analysis of the dependence of $\Omega \left(r\right)$ upon $\overline{c }\left(r\right)$ (Eq. (16)) with the activity coefficient ratio initially taken as unity (Wills et al. 1980). Refinement of the two activity coefficients on the basis of the resulting composition via Eq. (6) with respective values of ${B}_{ii}^{*}$ and ${B}_{ij}^{*}$ calculated from Eqs. (11) and (12) as well as third virial coefficients ${B}_{ijk}^{*}$ from counterparts of Eq. (19) was followed by reanalysis of the [$\overline{c }(r)$, ${{M}_{1}z}_{1}\left(r\right)$] data set to obtain revised estimates of ${X}_{2}^{app}$ and ${X}_{3}^{app}$ in an iterative process that was continued until no further change occurred in successive estimates of the two equilibrium constants.

**Fig. 2:**
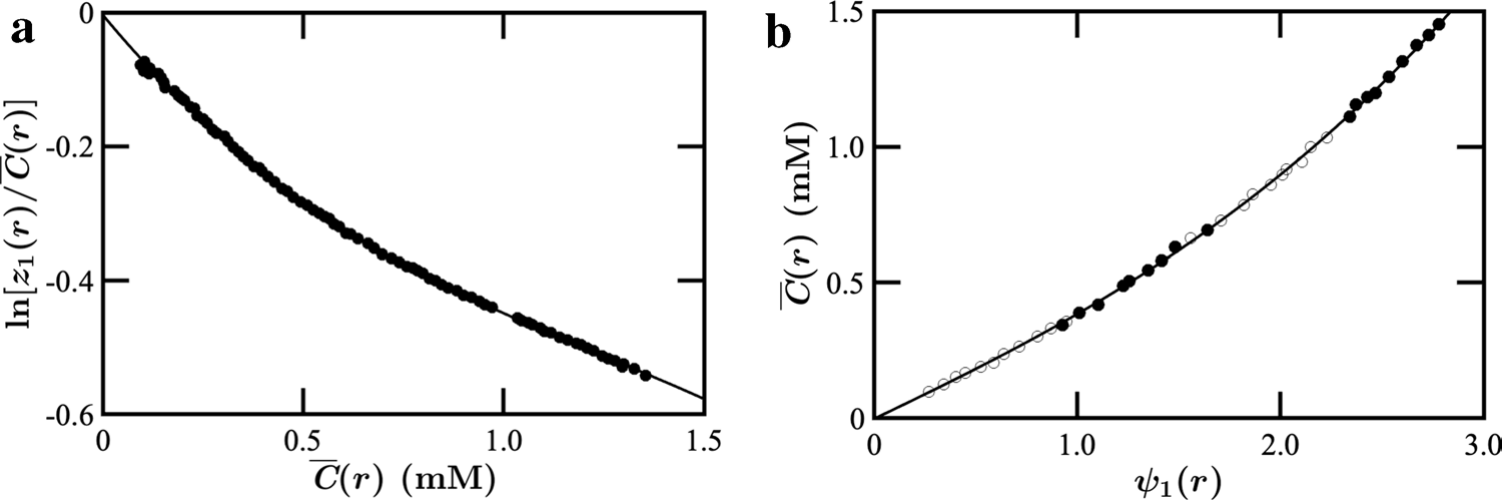
Characterization of lysozyme self-association by the original and modified Hill − Chen approaches. **a** Analysis of the [*r*, $\overline{c }(r)]$ data sets from four separate sedimentation equilibrium experiments (Wills et al. 1980) according to Eq. (17). **b** Corresponding analysis in terms of Eq. (26)

Interest in such endeavours only resumed after the demonstration (Wills and Winzor 1992; Wills et al. 1993) that the thermodynamic activity being monitored in sedimentation equilibrium experiments is, indeed, a molar quantity. Reanalyses of the same sedimentation equilibrium distributions for lysozyme according to the Hill-Chen procedure (Eq. (17)) (Wills and Winzor 1992) and its modified form (Eq. (26)) (Wills et al. 1996) are presented in Fig. 2a and b respectively. Nonlinear least squares curve-fitting of both data sets to a cubic polynomial yielded different estimates of ${K}_{2}$ and ${K}_{3}$. In view of the necessarily slower convergence of the series in Eq. (17), the values inferred from the fit shown in Fig. 2b are considered to be the more reliable estimates: ${z}_{1}({r}_{F})$ = 0.272 (± 0.003) mM for $\overline{c }({r}_{F})$ = 0.338 mM; ${b}_{2}=475\; (\pm 32)$ ${\mathrm{M}}^{-1}$; ${b}_{3}=$ 20,000 (± 13,000) M^−2^. Combination of the estimate of ${b}_{2}$ with the calculated value (Eq. (11) truncated at the second term) of 110 L/mol for ${B}_{11}^{*}$ in Eq. (23d) signifies a dimerization constant ${K}_{2}$ of 585 (± 32) M^−1^. On the basis of the estimate of 204,000 (± 24,000) M^−2^ for ${K}_{3},$ obtained from Eq. (23e) with ${B}_{12}^{*}$= 340 L/mol and ${B}_{111}^{*}$= 6100 L^2^ mol^−2^, the stepwise association constant for the formation of trimer from dimer (${K}_{3}/{K}_{2}$) is 350 (± 60) M^−1^. Although this result seemingly questions the validity of describing results for lysozyme in terms of indefinite self-association governed by a single stepwise (isodesmic) binding constant (Wills et al. 1980), it may well reflect inability of the nonlinear curve-fitting process to provide a definitive value for the fourth virial coefficient (${b}_{4}$)—a parameter whose delineation may well have led to consequent changes in the estimates of *b*_2_ and *b*_3_.

### Studies of α-chymotrypsin dimerization

The dimerization of α-chymotrypsin under mildly acidic conditions (pH 4) has figured prominently as a model system in tests of procedures for the characterization of protein self-association by sedimentation equilibrium (Winzor and Scheraga 1964; Morimoto and Kegeles 1967; Aune and Timasheff 1971; Aune et al. 1971; Horbett and Teller 1973, 1974; Gorbunoff et al. 1978). It also featured in an early application of the Omega procedure for direct analysis of sedimentation equilibrium concentration distributions (Tellam et al. 1979), where thermodynamic ideality was assumed to justify identification of the thermodynamic activity of monomer, ${M}_{1}{z}_{1}({r}_{F})$, obtained from *Ω*_*o*_ (Eq. (15)) with ${c}_{1}\left({r}_{F}\right)$, its corresponding weight-concentration ${c}_{1}(r)$ was calculated throughout the distribution as ${c}_{1}\left({r}_{F}\right){\psi }_{1}(r)$ then allowed generation of the corresponding dimer concentration distribution, ${c}_{2}$(r) = $[\overline{c }\left(r\right)-{c}_{1}\left(r\right)]$, and hence evaluation of the apparent association constant,${X}_{2}^{app}={c}_{2}(r)/[{c}_{1}(r){]}^{2}$, for each experimental [${c}_{1}\left(r\right), {c}_{2}\left(r\right)]$ combination. An apparent dimerization constant of 3.5 (± 0.1) L/g, or 4.4 (± 1.3) × 10^4^ M^−1^ was obtained by this procedure for α-chymotrypsin in acetate-chloride buffer, pH 3.9, *I*_*M*_ 0.2 (Tellam et al. 1979).

In the first investigation to employ ${\psi }_{1}\left(r\right)$ as the abscissa in allowance for the effects of thermodynamic nonideality on the statistical-mechanical basis of excluded volume, Eq. (4b) for a monomer–dimer system was rearranged as. 

28 $$\frac{\overline{c }\left(r\right){\gamma }_{1}(r)}{{\psi }_{1}(r)}={{M}_{1}z}_{1}\left({r}_{F}\right)+{X}_{2}[{M}_{1}{z}_{1}({r}_{F}){]}^{2}{\psi }_{1}(r)\left[\frac{{\gamma }_{1}(r)}{{\gamma }_{2}(r)}\right]$$

to allow determination of the dimerization constant from the linear dependence of $\overline{c }\left(r\right){\upgamma }_{1}(r)/{\psi }_{1}(r)$ upon ${\psi }_{1}\left(r\right){[\gamma }_{1}\left(r\right)/{\gamma }_{2}(r)].$ Its equivalence with the expression (Jacobsen and Winzor 1992)

29 $$\Omega \left(r\right){\gamma }_{1}(r)={\Omega }_{0}+{X}_{2}{\Omega }_{o}^{2}\{\overline c\left(r_F\right)\Omega_1(r)\}\left[\frac{{\gamma }_{1}(r)}{{\gamma }_{2}(r)}\right]$$

follows from substitution of the current Eqs. (14) and (15) for $\Omega \left(r\right)$ and ${\Omega }_{o}$ respectively. Equation (28) may be readily rearranged (Wills et al. 2012) as

30 $$\overline{c }\left(r\right)= \frac{{z}_{1}\left({r}_{F}\right){\psi }_{1}(r)}{{\gamma }_{1}(r)}+\frac{{X}_{2}{M}_{1}[{z}_{1}({r}_{F}){\psi }_{1}(r){]}^{2}}{{\gamma }_{2}(r)}$$

which is a simpler form suitable for the evaluation of ${z}_{1}({r}_{F})$ and the dimerization constant ${X}_{2}$ as curve-fitting parameters emerging from nonlinear regression analysis of the dependence of $\overline{c }\left(r\right)$ upon ${\psi }_{1}(r)$.

The activity coefficients (see Eq. (6)) are defined by the expressions

31a $${\gamma }_{1}\left(r\right)=\mathrm{exp}[2{B}_{11}^{*}{C}_{1}\left(r\right)+ {B}_{12}^{*}{C}_{2}\left(r\right\}].$$

31b $${\gamma }_{2}\left(r\right)=\mathrm{exp}[2{B}_{22}^{*}{C}_{2}\left(r\right)+ {B}_{12}^{*}{C}_{1}\left(r\right)]$$

in which values of the three second virial coefficients can be calculated from Eqs. (11) and (12) after assigning magnitudes to the radius $${R}_{i}$$ and net charge $${Z}_{i}$$ of both species. To that end, values of 2.44 nm and 3.07 nm were ascribed to $${R}_{1}$$ and $${R}_{2}$$ respectively on the basis of the Stokes radius of monomer and spherical geometry ($${R}_{2}={2}^{1/3}{R}_{1})$$; and the respective net charges of α-chymotrypsin monomer and dimer at pH 4 were taken as + 10 and + 20 (Ford and Winzor 1983).

**Fig. 3:**
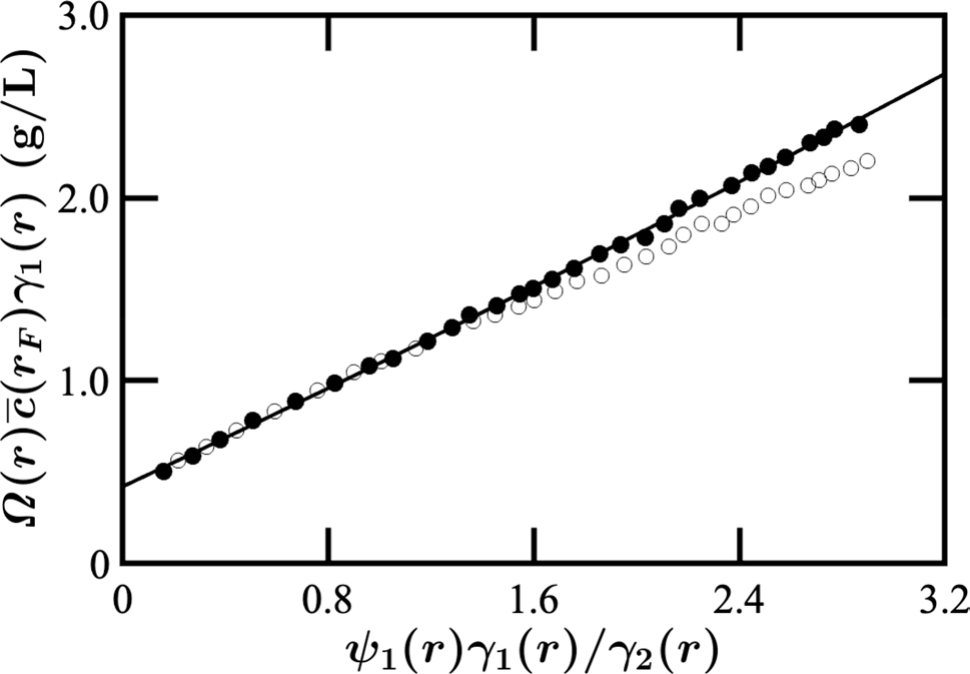
Iterative characterization of α-chymotrypsin dimerization (pH 3.9, *I*_*M*_ 0.2). Initial analysis of sedimentation equilibrium distributions according to Eq. (28) on the basis of thermodynamic ideality [${\gamma }_{1}(r)$ = ${\gamma }_{2}(r)$ = 1 for all *r*]. Revised analysis incorporating values of activity coefficients obtained from Eq. (31a, b) and the initial estimates of ${C}_{1}(r)$ and ${C}_{2}(r)$ deduced from the initial analysis. (Data taken from Fig. 2 of Jacobsen and Winzor 1992)

Composition dependence of the two activity coefficients necessitated the adoption of an iterative approach in which analysis of the experimental concentration distribution on the basis of thermodynamic ideality (◯, Fig. 3) was used to obtain values of ${c}_{1}(r)$ and ${c}_{2}(r)$ from the first estimate of ${{M}_{1}z}_{1}\left({r}_{F}\right)$, taken as ${c}_{1}\left({r}_{F}\right);$ and hence estimates of ${\gamma }_{1}(r)$ and ${\gamma }_{2}(r)$ throughout the distribution. Reanalysis of the experimental concentration distribution in terms of Eq. (28) then yielded an essentially linear dependence (⬤, Fig. 3) from which a dimerization constant of 3.7 (± 0.8) L/g or 4.6 (± 1.0) × 10^4^ M^−1^ was deduced (Jacobsen and Winzor 1992). Comparison of this value with the above estimate based on Omega analysis and assumed thermodynamic ideality establishes the relative unimportance of making allowance for effects of nonideality on the system at this moderately high ionic strength (*I*_*M*_ 0.2).

**Fig. 4:**
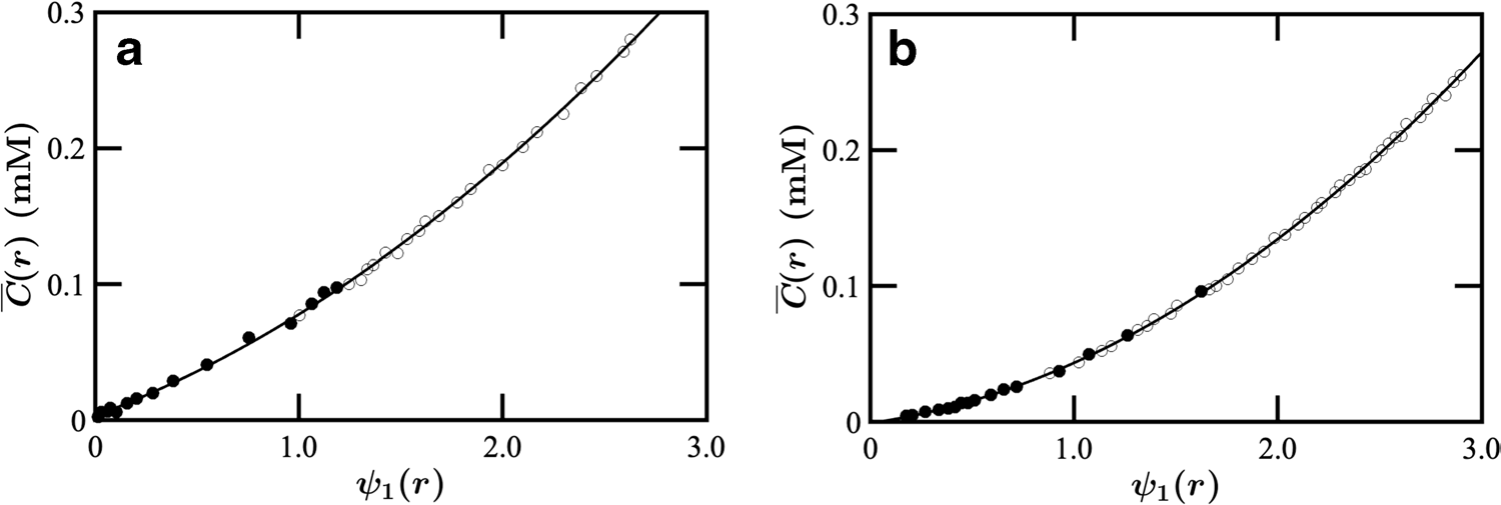
Characterization of the dimerization of α-chymotrypsin by the modified Hill − Chen procedure. a Analysis of sedimentation equilibrium distributions according to Eq. (24) truncated at the quadratic term for enzyme in acetate − chloride buffer (pH 4.1, ${I}_{M}$ 0.08). **b** Corresponding analysis of concentration distributions for enzyme under the conditions (pH 3.9, ${I}_{M}$ 0.2) pertaining to Fig. 3. (Data taken from Fig. 1 of Wills et al. 1996.)

In the first attempt to avoid the need for iterative analysis by adopting the modified Hill − Chen approach (Wills et al. 1996), the ionic strength was lowered to 0.08 M to enhance the relative influence of thermodynamic nonideality by decreasing the strength of α-chymotrypsin dimerization, for which the dominant effect of *I*_*M*_ is the change in general electrostatic repulsion (Agapow and Winzor 1988). Figure 4a summarizes the application of Eq. (24) to results from a high-speed sedimentation equilibrium experiment with an upper concentration limit of 2.2 g/L (⬤) and those (◯) from a low-speed run (1.8 < $\overline{c }(r)$ < 7.0 g/L), which were combined by selecting the reference radial position in each run on the basis of a common $\overline{c }({r}_{F})$ of 1.94 g/L. Because attempts to include the cubic term of Eq. (24) in the nonlinear curve-fitting procedure led to an unacceptable (positive) magnitude for the cubic coefficient ${b}_{3}$, which is necessarily negative for a monomer − dimer system, the analysis was repeated on the basis of Eq. (24) truncated at the quadratic term to yield a best-fit description (^_____^, Fig. 4a) signifying respective magnitudes of 59.2 (± 0.5) µM and 2510 (± 80) L/mol for ${z}_{1}({r}_{F})$ and $({K}_{2}- {B}_{11}^{*})$. A dimerization constant of 2820 (± 80) µM^−1^ was therefore deduced on the basis of the second virial coefficient ${B}_{11}^{*}$ of 309 L/mol that is calculated from Eq. (11) truncated at the second term. That value of ${K}_{2}$ increases to 2560 (± 80) after revising the magnitude of ${B}_{11}^{*}$ by including the final term in Eq. (11).

Cause for concern about the reliability of the above association constant stems from the corresponding analysis (Fig. 4b) of the dependence of $\overline{C }(r)$ upon ${\psi }_{1}\left(r\right)$ from sedimentation equilibrium distributions at the higher ionic strength (0.2 M) employed in Fig. 2. Continued failure of non-linear least-squares curve-fitting to Eq. (24) to provide an acceptable magnitude of ${b}_{3}$ again necessitated its truncation at the quadratic term—an action which led to a ${K}_{2}$ estimate of 3.0 (± 0.1) × 10^4^ M^−1^ that is considerably smaller than the previous estimate of 4.6 × 10^4^ M^−1^ obtained by allowance for nonideality contributions from the equivalent of cubic and quartic terms in Eq. (24). Proof of the need to obtain a physically acceptable estimate of ${b}_{3}$ comes from subsequent sedimentation equilibrium studies at even lower ionic strength (Wills and Winzor 2001; Wills et al. 2012).

**Fig. 5:**
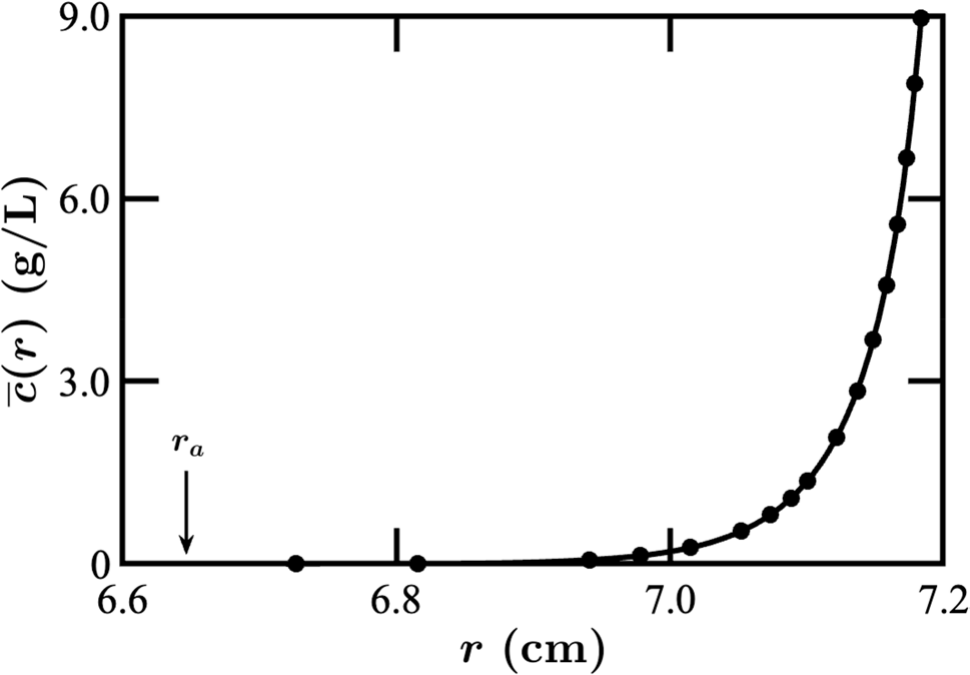
Concentration distribution from a high-speed sedimentation equilibrium experiment in a Beckman XL-I instrument (30,000 rpm, 293 K) on α-chymotrypsin in acetate − chloride buffer (pH 4.1, ${I}_{M}$ 0.05). ${r}_{a}$ denotes the position of the air − liquid meniscus. (Data inferred from Fig. 2 of Wills and Winzor 2001).

**Fig. 6:**
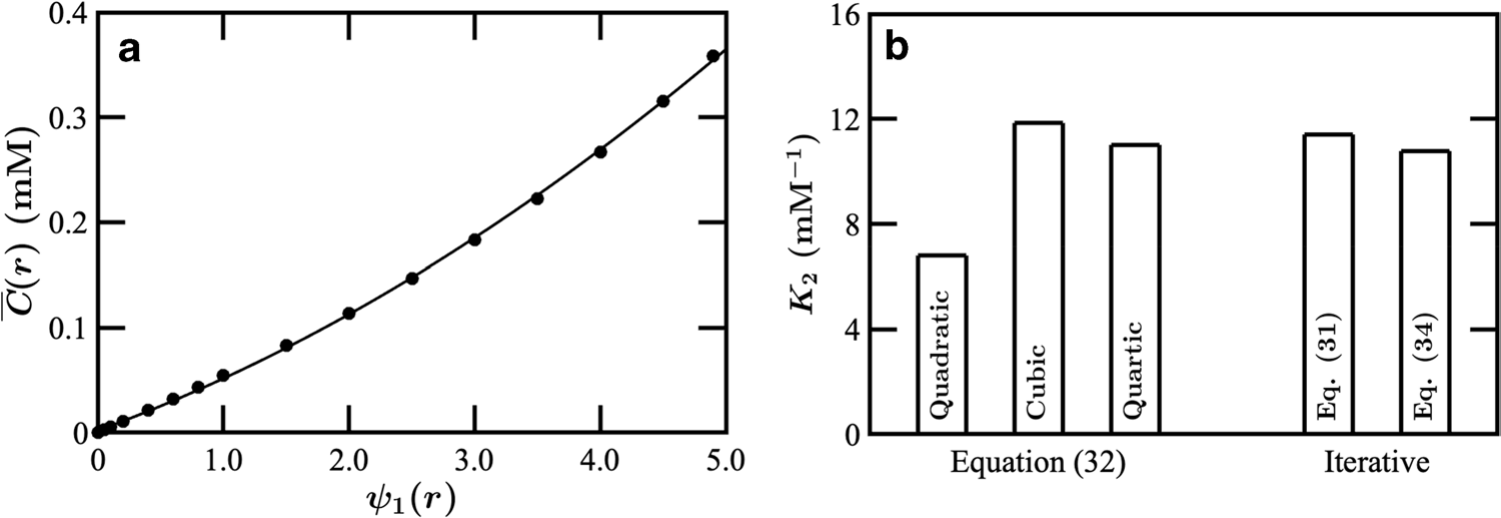
Evaluation of the dimerization constant for α-chymotrypsin in acetate − chloride buffer (pH 4.1, *I*_*M*_ 0.05) by direct analysis of the high-speed sedimentation equilibrium distribution shown in Fig. 5. **a** Dependence of $\overline{C }(r)$ upon ${\psi }_{1}(r)$ deduced from Fig. 5. **b** Estimates of ${K}_{2}$ based on Eq. (32) and its truncated forms, as well as those obtained by iterative analysis via Eq. (30) with activity coefficients calculated from Eqs. (31a, 31b) and (34a, 34b). (Data taken from Wills and Winzor 2001; Wills et al. 2012)

Whereas the above studies of the effect of thermodynamic nonideality on α-chyymotypsin dimerization have entailed the combination of low-speed and meniscus-depletion experiments to cover a sufficiently wide range of protein concentration $\overline{c }(r)$, the switch to a Beckman XL-I analytical ultracentrifuge for a subsequent study (Wills and Winzor 2001) of the enzyme at lower ionic strength (pH 4.1, *I*_*M*_ 0.05) rendered possible the collection of [$r, \overline{c }(r)]$ over the range 0 ≤ $\overline{c }(r)$ ≤ 8.4 g/L from a single high-speed sedimentation equilibrium run at 30,000 rpm (Fig. 5). Conversion of the radial distribution to one in terms of the Psi-function with ${r}_{F}=7.100$ cm is shown in Fig. 6a, where the number of experimental points (440) precludes any opportunity for visual discrimination between best-fit descriptions obtained by nonlinear least-squares curve-fitting to expressions such as Eq. (24). In that regard, the series has been extended to include a term to the quartic power of ${z}_{1}({r}_{F})$, whereupon the expression for total base-molar concentration, $\overline{C }(r)$, becomes (Wills et al. 2012)

32 $$\begin{array}{c}\overline C\left(r\right){=z_1\left(r_F\right)\psi}_1\left(r\right)+2\left(K_2-B_{11}^\ast\right)\lbrack{z_1\left(r_F\right)\psi}_1\left(r\right)\rbrack^2\\-3\{2(B_{11}^\ast)^2-B_{111}^\ast/2-K_2B_{12}^\ast\}\lbrack{z_1\left(r_F\right)\psi}_1\left(r\right)\rbrack^3\\+4\{3B_{11}^\ast B_{111}^\ast-16\lbrack(B_{11}^\ast)^3+B_{1111}^\ast/3\rbrack+K_2\lbrack2B_{11}^\ast B_{12}^\ast+\{(B_{12}^\ast)^2-B_{112}^\ast\}/2\rbrack\\-K_2^2B_{22}^\ast{\}\lbrack z_1\left(r_F\right)\psi}_1\left(r\right)\rbrack^4+...\end{array}$$

A magnitude for the fourth virial coefficient for monomer self-interaction (${B}_{1111}^{*}$) is approximated by the expression

33a $${B}_{1111}^{*}=1175.35{\pi }^{3}{N}_{A}^{3}({R}_{1}^{eff}{)}^{3}/27$$

33b $$R_1^{eff}=\sqrt[3]{3B_{11}^\ast/(16\pi N_A)}\;$$

which is that for an uncharged sphere (Blaak 1998) with ${R}_{1}^{eff}$ the effective monomer radius of an uncharged sphere with the same second virial coefficient (Minton and Edelhoch 1982). Reliable values for all of the other virial coefficients require determination by numerical integration at this low ionic strength: ${B}_{11}^{*},{B}_{12}^{*} , \mathrm{and}\;{B}_{22}^{*}$ from Eq. (8); and ${B}_{111}^{*}$ from Eq. (19), the right-hand side of which can also be used to cover the interaction between two monomer molecules (*i*, *j*) and a dimer molecule (*k*) to obtain ${B}_{112}^{*}$ (Wills et al. 2012).

A major finding to emerge from analysis of the dependence (Fig. 6a) of $\overline{C }(r)$ upon ${\psi }_{1}(r)$ in terms of Eq. (32) is the need for extension of the nonlinear least-squares curve-fitting to at least the cubic term in order to obtain a reliable estimate of the dimerization constant. Truncation of that curve-fitting at the quadratic term leads to an estimate of 680 (± 16) M^−1^ for ${K}_{2}$, which increases substantially to 1185 (± 7) M^−1^ when the cubic term is included in the curve-fitting process (Fig. 6b). Little change in the estimated magnitude of the dimerization constant [${K}_{2}$= 1101 (± 10) M^−1^] results from extension of series to the quartic term. An important corollary to this observation is that the modified Hill-Chen analysis is limited to the characterization of reversible dimerization because of the need for meaningful quantification of the series in Eq. (32) to higher orders in monomer thermodynamic activity in order to obtain a reliable estimate of ${K}_{2}$ (Wills and Winzor 2001; Scott et al. 2010; Wills et al. 2012).

We therefore return to the more generally applicable practice of employing iterative analysis to evaluate values of composition-dependent activity coefficients and hence ${K}_{2}$ from the molar counterpart of Eq. (30) with extended counterparts of Eqs. (31a) and (31b), namely

34a $${\gamma }_{1}\left(r\right)=\mathrm{exp}\left[\begin{array}{c}2{B}_{11}^{*}{C}_{1}\left(r\right)+{B}_{12}^{*}{C}_{2}\left(r\right)+\frac{3}{2}{B}_{111}^{*}\{{C}_{1}\left(r\right){\}}^{2}+{B}_{112}^{*}{C}_{1}\left(r\right){C}_{2}\left(r\right)\\ \\ +\frac{4}{3}{B}_{1111}^{*}\{{C}_{1}\left(r\right){\}}^{3}+\dots \end{array}\right]$$

34b $${\gamma }_{2}\left(r\right)=\mathrm{exp}\left[2{B}_{22}^{*}{C}_{2}\left(r\right)+{B}_{12}^{*}{C}_{1}\left(r\right)+\frac{1}{2}{B}_{112}^{*}\{{C}_{1}\left(r\right){\}}^{2}+{B}_{122}^{*}{C}_{1}\left(r\right){C}_{2}\left(r\right) \dots \right]$$

that include additional thermodynamic nonideality contributions arising from self-interaction of three and four monomers (the ${B}_{111}^{*}$ and ${B}_{1111}^{*}$ terms) as well as those involving physical interaction between two molecules of species *i* and one of species *j* (the ${B}_{112}^{*}$ and ${B}_{122}^{*}$ terms). From Fig. 6b, it is evident that the simpler allowance for thermodynamic nonideality on the basis of nearest-neighbor interactions (Eqs. (31a, b)) suffices to yield a dimerization constant, 1141 (± 13) M^−1^, that is flanked by the two estimates obtained by the modified Hill-Chen approach (Eq. (24)). Furthermore, the inclusion of higher-order terms in the activity coefficient expressions Eqs. (34a, 34b) had a relatively minor effect on the estimate (1078 (± 10) M^−1^) of ${K}_{2}$.

**Fig. 7:**
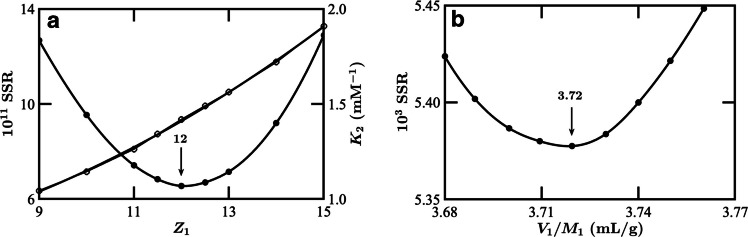
Test of the feasibility of incorporating the consequences of monomer net charge as an additional curve-fitting parameter in the analysis of self-associating systems. **a** Evaluation of ${Z}_{1}$ as an additional parameter on the basis of the SSR (⬤) associated with the best-fit descriptions of the $\overline{C }\left(r\right)\;vs\;{\psi }_{1}(r)$ dependence (Fig. 6a) in terms of Eq. (24) and virial coefficient estimates from Eq. (23d, 23e). The corresponding best-fit value ${K}_{2}$ for each assigned value of ${Z}_{1}$ is also shown (◯). (Data taken from Fig. 2 of Wills et al. 2012) **b** Corresponding use of the SSR to locate the best-fit value of the solvated monomer specific volume $({V}_{1}/{M}_{1})$, the parameter employed in SPT to deduce species activity coefficients by iterative application of Eqs. (36a-36c) and Eq. (35) by analysis of a simulated sedimentation equilibrium distribution (Eq. (32)) for a monomer‒tetramer system $({M}_{1}$= 66 kDa, ${R}_{1}$= 3.5 nm, ${X}_{4}$ = 0.56 ${\mathrm{L}}^{3}{\mathrm{g}}^{-3}$, ${I}_{M}$ = 0.05). (Data taken from Fig. 2 of Scott et al. 2010)

All of the analyses summarized in Fig. 6b entail the incorporation of a monomer net charge (${Z}_{1}$) of + 10 into the calculation of virial coefficients for α-chymotrypsin at pH 4.1, this being an experimental value deduced (at pH 3.9) from measurement of the Donnan distribution of ions in equilibrium dialysis (Ford and Winzor 1983). Also examined (Wills et al. 2012) was the possibility that ${Z}_{1}$ could be regarded as an additional parameter to evolve from the best-fit description of the transformed experimental concentration distribution (Fig. 6a) in terms of Eq. (30) and activity coefficients based on Eqs. (31a, 31b). The degree of conformity, as measured by the sum-of-squares of residuals (SSR) between the experimental data (Fig. 6a) and their best-fit description in terms of Eqs. (30) and (31a, 31b) for a range of ${Z}_{1}$, is shown (⬤) in Fig. 7a, which also includes the corresponding estimates of ${K}_{2}$ (◯). In that regard, the optimal monomer valence of 12 inferred from the minimum SSR exceeds the value of + 11 that is deduced from the amino acid composition and pH titration data for α-chymotrypsin (Marini and Wunsch 1963).

The findings presented in Fig. 7a serve to emphasize that the uncertainty estimate associated with a measured association constant merely refers to its precision. Although the precision (± 2SD) of each *K*_2_ in Fig. 6b is about 1%, its accuracy depends upon the magnitude assigned to *Z*_1_. A greater obstacle to better characterization of protein self-association is therefore likely to be the lack of a definitive estimate of monomer net charge, an important but rarely measured parameter (Winzor 2004). The monomer net charge has a more pronounced effect on the measured equilibrium constant than any deficiency in current procedures for incorporating the effect of thermodynamic nonideality into the analysis of sedimentation equilibrium distributions reflecting reversible protein self-association.

### SPT approach to activity coefficient determination

Scaled particle theory (Lebowitz et al. 1955; Reiss et al. 1959) affords an alternative statistical-mechanical treatment of thermodynamic nonideality for uncharged species. Under those circumstances (all $${Z}_{i}=0)$$, the required virial coefficients and hence activity coefficients become functions of monomer molar volume, $${V}_{1}=4\pi {N}_{A}{R}_{1}^{3}/3$$. In an attempt to extend the scope of scaled particle theory (SPT) to sedimentation equilibrium studies of protein self-association (Chatelier and Minton 1987; Zorilla et al. 2004; Jiménez et al. 2007), the stance has been taken (Minton and Edelhoch 1982) that the additional excluded volume arising from charge-charge repulsion can be accommodated by increasing the effective size of the solute species. The effective monomer volume then becomes $${V}_{1}^{eff}$$= $$4\pi {N}_{A}({R}_{1}^{eff}{)}^{3}/3$$ and the corresponding parameter for a polymer containing *n* monomers is expressed as $${V}_{n}^{eff}=n{x}^{3}{V}_{1}^{eff}$$ to allow for departures from volume conservation. $${V}_{1}^{eff}$$ and $${x}^{3}$$ then become additional curve-fitting parameters (as well as $${z}_{1}({r}_{F})$$ and $${X}_{n})$$ to emanate from iterative analysis of a sedimentation equilibrium distribution in terms of the general counterpart of Eq. (30),

35 $$\overline{c }\left(r\right)=\frac{{M}_{1}{z}_{1}\left({r}_{F}\right){\psi }_{1}\left(r\right)}{{\gamma }_{1}(r)}+\frac{{X}_{n}[{M}_{1}{z}_{1}\left({r}_{F}\right){\psi }_{1}\left(r\right){]}^{n}}{{\gamma }_{n}(r)}$$

in which the initial estimates of the two activity coefficients are obtained from the values of ${c}_{1}\left(r\right)={z}_{1}(r)$ deduced from the apparent association constant ${X}_{n}^{app}$ based on thermodynamic ideality. Those activity coefficients for monomer and *n*-mer are then calculated from the relationships (Gibbons 1969; Chatelier and Minton 1987; Scott and Winzor 2009)

36a $$Q=1-\left(\frac{{V}_{1}^{eff}}{{M}_{1}}\right){c}_{1}(r)+{x}^{3}\left[\overline{c }\left(r\right)-{c}_{1}\left(r\right)\right]$$

36b $$Q{\gamma }_{1}\left(r\right)={\mathrm{exp}}\left\{\begin{array}{c}\left(\frac{1}{Q}\right)\left(\frac{{V}_{1}^{eff}}{{M}_{1}}\right)\left[7{c}_{1}\left(r\right)+\left(\frac{{3x}^{2}}{{n}^{1/3}}+\frac{3x}{{n}^{2/3}}+\frac{1}{n}\right)\left[\overline{c }\left(r\right)-{c}_{1}\left(r\right)\right]\right]\\ +\left(\frac{3}{{Q}^{2}}\right){\left(\frac{{V}_{1}^{eff}}{{M}_{1}}\right)}^{2}\left[{c}_{1}\left(r\right)+\frac{{x}^{2}\left[\overline{c }\left(r\right)-{c}_{1}\left(r\right)\right]}{{n}^{1/3}}\right]\\ \times \left[\frac{5}{2}{c}_{1}\left(r\right)+\left(\frac{{3x}^{2}}{2{n}^{1/3}}+\frac{x}{{n}^{2/3}}\right)\left[\overline{c }\left(r\right)-{c}_{1}\left(r\right)\right]\right]\\ +\left(\frac{3}{{Q}^{3}}\right){\left(\frac{{V}_{1}^{eff}}{{M}_{1}}\right)}^{3}{\left[{c}_{1}\left(r\right)+\frac{{x}^{3}\left[\overline{c }\left(r\right)-{c}_{1}\left(r\right)\right]}{{n}^{1/3}}\right]}^{3}\end{array}\right\}$$

36c $$Q{\gamma }_{n}(r)={\mathrm{exp}}\left\{\begin{array}{c}\left(\frac{1}{Q}\right)\left(\frac{{V}_{1}^{eff}}{{M}_{1}}\right)\left[7{x}^{3}\left[\overline{c }\left(r\right)-{c}_{1}\left(r\right)\right]+\left(3{n}^{1/3}x+3{n}^{2/3}{x}^{2}+n{x}^{3}\right)\left[{c}_{1}\left(r\right)\right]\right]\\ +\left(\frac{3}{{Q}^{2}}\right){\left(\frac{{V}_{1}^{eff}}{{M}_{1}}\right)}^{2}\left[{x}^{3}\left[\overline{c }\left(r\right)-{c}_{1}\left(r\right)\right]+{n}^{1/3}x{c}_{1}(r)\right]\\ \times \left[5{x}^{3}\left[\overline{c }\left(r\right)-{c}_{1}\left(r\right)\right]/2 +\left(3{n}^{1/3}x/2+{n}^{2/3}{x}^{2}\right){c}_{1}(r)\right]\\ +\left(\frac{3}{{Q}^{3}}\right){\left(\frac{{V}_{1}^{eff}}{{M}_{1}}\right)}^{3}{\left[{x}^{3}\left[\overline{c }\left(r\right)-{c}_{1}\left(r\right)\right]+{n}^{1/3}x{c}_{1}(r)\right]}^{3}\end{array}\right\}$$

Sedimentation equilibrium distributions simulated on the basis of the potential-of-mean-force model (McMillan and Mayer 1945) have been used (Scott et al. 2010) to test the efficacy of this SPT approach to characterizing the self-association of a charged protein. Those simulations referred to monomer-tetramer systems involving a 66-kDa monomer with a radius of 3.5 nm and a net charge of 20 in buffer media with ionic strengths of 0.05 M and 0.15 M. The three virial coefficients (${B}_{11}^{*}$, ${B}_{12}^{*}$ and ${B}_{22}^{*}$) required for calculation of the two activity coefficients, ${\gamma }_{1}(r)$ and ${\gamma }_{4}(r)$ in Eq. (35), were obtained by numerical integration of Eq. (8a). Here we focus attention on the simulated concentration distribution (15,000 rpm and 20 °C with ${c}_{1}\left({r}_{F}\right)=1.5$ g/L in a liquid column spanning the range 6.85 ≤ *r*$\le$ 7.15 cm) at the lower ionic strength for a system with ${X}_{4}$ = 0.56 × 10^−4^ L^3^ g^−3^ (${K}_{4}$ = 1.61 × 10^10^ M^−3^).

The SSR associated with best-fit descriptions of the simulated sedimentation equilibrium distribution in terms of Eq. (35) with activity coefficients obtained from Eqs. (36a-36c) with ${x}^{3}$ = 1 and assigned values of ${V}_{1}^{eff}/{M}_{1}$ (Scott et al. 2010) are shown in Fig. 7b. On that basis, the most appropriate magnitude for ${V}_{1}^{eff}/{M}_{1}$ is 3.72 mL/g, which signifies an effective second virrial coefficient for monomer [${B}_{11}^{eff}=4{M}_{1}({V}_{1}^{eff}{)}_{min}$] of 982 L/mol that agrees reasonably well with the input value of 1020 L/mol for ${B}_{11}^{*}$. The corresponding estimate of 0.61 (± 0.02) × 10^−4 ^${\mathrm{L}}^{3}{\mathrm{g}}^{-3}$ for ${X}_{4}$ differs from the input value of 0.56 × 10^−4 ^${\mathrm{L}}^{3}{\mathrm{g}}^{-3}$ by 9 (± 4)%. Although pronounced improvement in the fit could be effected by allowing the value of ${x}^{3}$ to float with ${V}_{1}^{eff}/{M}_{1}$ fixed, the return of values substantially below unity is inconsistent with unity being a realistic lower limit for the relative size parameter *x*.

This application of SPT to characterizing nonideal self-association from a simulated sedimentation equilibrium distribution for a charged protein serves to establish the likely adequacy of an association constant for a specified reaction stoichiometry. However, the two size-related quantities are merely empirical curve-fitting parameters that may bear little relationship to the physical characteristics of the species that they supposedly characterize (Scott et al. 2010). The conventional statistical-mechanical approach (McMillan and Mayer 1945) adopted throughout this treatise is therefore considered to be the more reliable procedure for quantifying the self-association of charged proteins by direct analysis of sedimentation equilibrium distributions.

### Protein self-association in the presence of small cosolutes

An enhanced extent of protein self-association is likely to occur in plants and other organisms that generate high concentrations of small solutes (osmolytes) such as glucose, sucrose, betaine, and proline in response to biological stress (Paleg and Aspinall 1981). In these molecular crowding situations where the concentration of cosolute, ${C}_{M}$, greatly exceeds that ($\overline{C }$) of a reversibly dimerizing protein, the expressions for the monomer and dimer activity coefficients (see Eq. (6)) become dominated by the second virial coefficient terms for interaction between cosolute and the two solute species (Nichol et al. 1981), whereupon the measured equilibrium constant is related to its thermodynamic counterpart ${K}_{2}$ by the expression

37 $${(K}_{2}^{app}{)}_{M} = {C}_{2}/{C}_{1}^{2} \approx {K}_{2}\mathrm{exp}[\left(2{B}_{1M}^{*}-{B}_{2M}^{*}\right){C}_{M}]$$

**Fig. 8:**
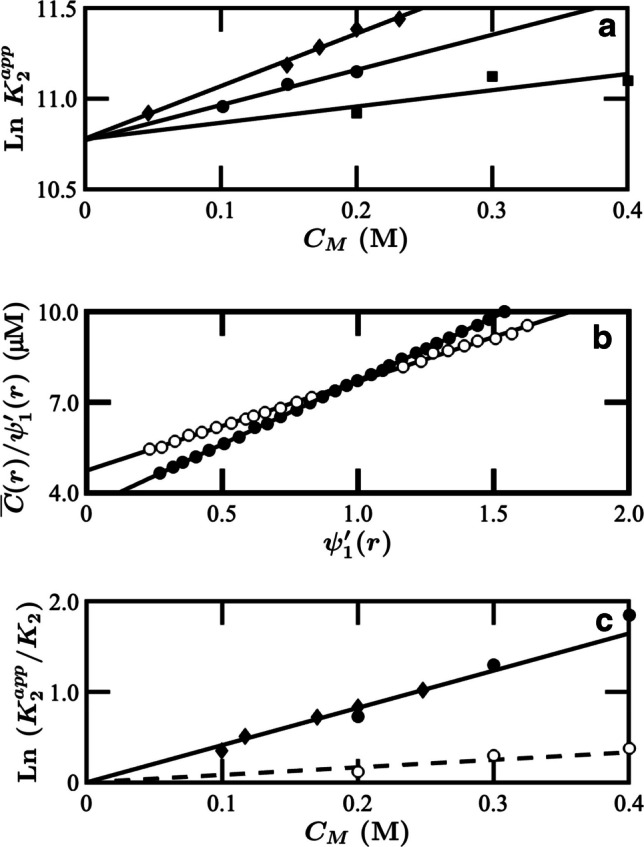
Enhancement of the α-chymotrypsin dimerization by small inert cosolutes (*M*) in acetate-chloride buffer (pH 3.9, ${I}_{M}$ 0.2). **a** Reported dependence of the apparent association equilibrium constant upon *C*_*M*_ for glucose (◼), sucrose (⬤) and raffinose (◆). (Data taken from Fig. 2 of Patel et al. (2002)). **b** Disparity between the Psi analysis (◯) according to Eq. (38) with monomer buoyant molecular mass ${M}_{b }$ miscalculated as ${M}_{1}\left(1-\overline{v}{\rho }_{d}\right)$ from the density ${\rho }_{d}$ of buffer supplemented with 0.2 M sucrose and that (⬤) in which Eq. (40) was used for ${M}_{b}$. (Data inferred from Fig. 1 of Winzor et al. (2007a)). **c** Conformity of the reassessed dependence of ${K}_{2}^{app}$ for the dimerization of α-chymotrypsin upon sucrose concentration (⬤,◆) with the behaviour (^**_____**^) predicted by Eq. (41); and (^**---**^), the corresponding dependence (Patel et al. 2002) based on the incorrect value for monomer buoyant molecular mass

Admittedly, the magnitude of the covolume difference $$\left(2{B}_{1M}^{*}-{B}_{2M}^{*}\right)$$ in Eq. (37) is small for these osmolytes, but should be offset by the large cosolute concentrations (> 0.1 M) that are generated in response to biological stress. Sedimentation equilibrium studies of α-chymotrypsin dimerization (pH 3.9, $${I}_{M}\;0.2)$$ in the presence of saccharides (Shearwin and Winzor 1988; Patel et al. 2002; Winzor et al. 2007a) have substantiated that reasoning. Analysis of results (Patel et al. 2002) for enzyme in the presence of glucose (◼), sucrose (⬤), and raffinose (◆) exhibited the progressive increase in slope of the dependence of $$\mathrm{ln}{ K}_{2}^{app}$$ upon cosolute concentration with increasing saccharide size (Fig. 8a) as predicted by Eq. (37). However, the measured slopes were much smaller than the values of (2 $${B}_{1M}^{*}-{B}_{2M}^{*})$$ calculated from the first term of Eq. (12) with $${R}_{1}$$ = 2.44 nm, $${R}_{2}$$ = 3.07 nm (as above), and effective radii ($${R}_{M}$$) of 0.25 nm for glucose, 0.35 nm for sucrose, and 0.43 nm for raffinose. The fact that results from an earlier study (Shearwin and Winzor 1988) based on Omega analysis of distributions for the α-chymotrypsin-sucrose system had yielded a slope in conformity with Eq. (37) prompted closer scrutiny of the expressions used for the buoyant molecular mass in the calculations of $${\psi }_{1}\left(r\right).$$

For α-chymotrypsin in the absence of cosolute, the low enzyme concentration justifies the truncation of Eq. (32) at the linear term in monomer thermodynamic activity, whereupon

38 $$\begin{array}{c}\overline C\left(r\right)=z_1\left(r_F\right)\psi_1\left(r\right)+2\left(K_2-B_{11}^\ast\right)\lbrack z_1(r_F)\psi_1(r)\rbrack^2\\\approx z_1\left(r_F\right)\psi_1\left(r\right)+2K_2\lbrack z_1(r_F)\psi_1(r)\rbrack^2\end{array}\\$$

The assumption inherent in the approximate form is thus seen to be that ${K}_{2}\gg {B}_{11}^{*}$, which is certainly met for the present system with ${K}_{2}=\mathrm{46,000}$ L/mol (Fig. 3) and ${B}_{11}$ = 188 L/mol. Protein − cosolute mixtures for sedimentation equilibrium experiments were prepared by prior exhaustive dialysis against buffer supplemented with cosolute concentration ${C}_{M}$ in order to establish its free concentration in the mixture. Although some redistribution of cosolute *M* in accordance with Eq. (1) occurs during the attainment of sedimentation equilibrium, the extent of that redistribution for a small species is sufficiently slight to justify the approximation that the cosolute concentration is uniformly ${C}_{M}$ throughout the distribution. This allows the cosolute to be regarded as part of a solvent with revised density ${\rho }_{d}$.

The equilibrium distribution of monomer then becomes (Casassa and Eisenberg 1964; Jacobsen et al. 1996; Deszczynski et al. 2006)

39a $${z}_{1}\left(r\right)={z}_{1}\left({r}_{F}\right){\psi }_{1}^{ {\prime}}(r)$$

39b $${\psi }_{1}^{ {\prime}}(r)=\mathrm{exp}[{M}_{1}(1-{\phi }^{ {\prime}}{\rho }_{d}){\omega }^{2}({r}^{2}-{r}_{F}^{2})/(2RT)$$

39c $${\rho }_{d}={\rho }_{s}+(1- {\overline{v} }_{M}{\rho }_{s}){M}_{M}{C}_{M}$$

in which the buoyancy factor is described in terms of the cosolute-supplemented buffer density (${\rho }_{d}$) and the apparent partial specific volume of the protein ${(\phi }^{ {\prime}})$. This revised buoyant molecular mass is related to the usual parameter by the relationship (Jacobsen et al. 1996)

40 $${M}_{1}\left(1-{\phi }^{ {\prime}}{\rho }_{d}\right)={M}_{1}\left(1-\overline{v}{\rho }_{s}\right)-\left(1-{\overline{v} }_{M}{\rho }_{s}\right){B}_{1M}{M}_{M}{C}_{M}$$

which allows the calculation of ${\psi }_{1}^{ {\prime}}(r)$ throughout the sedimentation equilibrium distribution, and hence determination of the enhanced extent of dimerization by means of the expression

41 $$\overline{C }\left(r\right)={z}_{1}\left({r}_{F}\right){\psi }_{1}^{ {\prime}}(r)+2{K}_{2}^{app}[{z}_{1}({r}_{F}){\psi }_{1}^{ {\prime}}(r){]}^{2} +\dots$$

as the counterpart of Eq. (38).

Reference to the two experimental studies of the effect of small cosolutes on sedimentation equilibrium distributions for α-chymotrypsin (Shearwin and Winzor 1988; Patel et al. 2002) shows that the buoyant molecular mass of monomer was calculated incorrectly as ${M}_{1}(1-\overline{v}{\rho }_{d})$, which introduces error (see Eq. (39b)) into the estimates of ${\psi }_{1}^{ {\prime}}(r).$ The consequences of this error are demonstrated in Fig. 8b, where the use of incorrect ${\psi }_{1}^{ {\prime}}(r)$ values (◯) clearly gives rise to a lower slope for the dependence of $\overline{C }(r)/{\psi }_{1}^{ {\prime}}(r)$ upon ${\psi }_{1}^{ {\prime}}(r)$ than that when the correct ${\psi }_{1}^{ {\prime}}(r)$ values (⬤) are used. The same error is necessarily incorporated into the Omega analysis (Shearwin and Winzor 1988) because of the definition of the ordinate parameter as $\Omega (r) =\overline{C }(r)/[\overline{C }({r}_{F}){\psi }_{1}^{ {\prime}}(r)]$. However, the consequent error in considering the ordinate intercept of the dependence of $\Omega \left(r\right)$ upon $\overline{C }\left(r\right)$ as ${z}_{1}\left({r}_{F}\right)/\overline{C }({r}_{F})$ self-cancels in the subsequent determination of ${z}_{1}(r)$ throughout the distribution as ${z}_{1}\left({r}_{F}\right){\psi }_{1}^{ {\prime}}(r)$. Correct values of ${K}_{2}^{app}$ were therefore obtained despite invalidity of the interpretation accorded by the Omega plot on which they were based (Winzor et al. 2007a).

This reappraisal of sedimentation equilibrium distributions for sucrose-supplemented α-chymotrypsin solutions in terms of the modified Psi approach (Patel et al. 2007a) is shown (⬤) in Fig. 8c, which also presents the original results (◯). This reanalysis has not only brought them into line with the earlier findings (Shearwin and Winzor 1988) based on Omega analysis (◆) but also into agreement with the theoretical dependence (^_____^) predicted by Eq. (37). The case for considering the effects of small cosolutes on protein self-association on the statistical-mechanical basis of excluded volume (molecular crowding) is thereby restored.

### Potential for model-independent identification of reaction stoichiometry

Delineation of the stoichiometry of protein self-association involving multiple oligomeric states has traditionally been based on the goodness-of-fit obtained by nonlinear least-squares analysis of dependencies of ${z}_{1}\left(r\right)$ upon $\overline{c }(r)$ for various models of the self-association process. A limitation of that approach is an inability to extract reliable estimates of the polynomial coefficients in expressions such as Eq. (22) and Eq. (24)—a factor evident from the discussion of Fig. 6. Independent identification of the relevant oligomeric species would assist greatly in the quantitative characterization of the protein self-association by restricting the problem to allowance for effects of thermodynamic nonideality in a system with defined self-association stoichiometry. We therefore explore the potential of sedimentation equilibrium as a way of defining the reaction stoichiometry. In that regard, the situation will be simplified by restricting the upper limit of the sedimentation distributions to below 3 − 4 g/L, thereby rendering reasonable the approximation inherent in substitution of solute concentrations for their thermodynamic activities. The consequences of thermodynamic nonideality can then be addressed after the self-association stoichiometry has been identified.

With that approximation, the description of a sedimentation equilibrium distribution for a self-associating solute in terms of the Psi function (Eq. 4a) becomes

42a $$\overline{c }\left(r\right)={c}_{1}\left({r}_{F}\right){\psi }_{1}\left(r\right)+{c}_{2}\left({r}_{F}\right){\psi }_{2}\left(r\right)+{c}_{3}\left({r}_{F}\right){\psi }_{3}\left(r\right)+{c}_{4}\left({r}_{F}\right){\psi }_{4}(\mathrm{r}) +\dots$$

42b $$= {c}_{1}\left({r}_{F}\right){\psi }_{1} \left(r\right)+{c}_{2}\left({r}_{F}\right){[\psi }_{1}\left(r\right){]}^{2}+{c}_{3}\left({r}_{F}\right){[\psi }_{1}\left(r\right){]}^{3}+{c}_{4}\left({r}_{F}\right)[{\psi }_{1}(r){]}^{4} +\dots$$

where $\overline{c }\left(r\right)$ is the total weight concentration of solute at radial distance *r*, and where the same partial specific volume is considered to apply to all oligomeric states of the solute. For present purposes, it is more convenient to divide Eq. (42b) by ${\psi }_{1}\left(r\right)$ to obtain the relationship

43 $$\overline{c }\left(r\right)/{\psi }_{1}(r)={c}_{1}\left({r}_{F}\right)+{c}_{2}\left({r}_{F}\right){\psi }_{1}(r)+{c}_{3}\left({r}_{F}\right)[{\psi }_{1}(r){]}^{2}+ {c}_{4}\left({r}_{F}\right)[{\psi }_{1}(r){]}^{3} + \dots$$

which signifies that the ordinate intercept of the dependence of $\overline{c }\left(r\right)/{\psi }_{1}(r)$ upon ${\psi }_{1}(r)$ defines ${c}_{1}\left({r}_{F}\right),$ the monomer concentration at the reference radial position: a value of zero for that intercept would imply the essential absence of monomer in the self-associating system. Knowledge of the value of ${c}_{1}\left({r}_{F}\right)$ then allows the calculation of monomer concentration throughout the distribution as ${c}_{1}\left(r\right)={c}_{1}\left({r}_{F}\right){\psi }_{1}\left(r\right).$ This approach for obtaining the concentration of monomer, ${c}_{1}(r)$, as a function of total solute concentration, $\overline{c }(r)$, without requiring knowledge of the self-association stoichiometry is, of course, analogous to defining the dependence on ${z}_{1}\left(r\right)$ upon $\overline{C }\left(r\right)$ in the characterization of nonideal self-association by the Omega procedure (Milthorpe et al. 1975; Wills et al. 1980).

Knowledge of ${c}_{1}(r)$ throughout the sedimentation equilibrium pattern allows the delineation of a residual total concentration distribution $\overline{c }^{\prime}(r)$, which, from Eq. (42b), may be written as

44 $${\overline{c} }{^{\prime}}\left(r\right)=\overline{c }\left(r\right)-{c}_{1}\left(r\right)={c}_{2}\left({r}_{F}\right){[\psi }_{1}\left(r\right){]}^{2}+{c}_{3}\left({r}_{F}\right){[\psi }_{1}\left(r\right){]}^{3}+{c}_{4}\left({r}_{F}\right)[{\psi }_{1}(r){]}^{4} +\dots$$

An expression analogous to Eq. (43) for the residual total concentration distribution, $\text{then becomes}$

45 $${\overline{c} }^{\prime}(r)/{\psi }_{2}\left(r\right)={\overline{c} }^{\prime}(r)/[{\psi }_{1}\left(r\right){]}^{2}={c}_{2}\left({r}_{F}\right)+{c}_{3}\left({r}_{F}\right){\psi }_{1}(r)+{c}_{4}\left({r}_{F}\right)[{\psi }_{1}(r){]}^{2}$$

from which an estimate of ${c}_{2}\left({r}_{F}\right)$ should be obtainable as the ordinate intercept of the dependence of ${\overline{c} }^{\prime}(r)/{\psi }_{2}\left(r\right)$ upon ${\psi }_{1}\left(r\right).$ Such evaluation of ${c}_{2}\left({r}_{F}\right)$ then provides the means of determining the concentration distribution for dimer, and hence of extracting a further residual concentration distribution, ${\overline{c} ^{\prime\prime}}(r)={\overline{c} }^{\prime}\left(r\right)-{c}_{2}(r)$ in which trimer becomes the smallest species.

In principle, this successive subtraction procedure has the potential to identify the concentration distributions for all participating species in the self-associating system. However, it must be recognized that the experimental uncertainty in ${c}_{1}({r}_{F})$ is incorporated into the estimated monomer distribution that is used to calculate the $\overline{c }{\prime}(r)$ distribution. This residual concentration is therefore delineated at a confidence level lower than its $\overline{c }(r)$ counterpart. Although this problem is exacerbated with each successive determination of a residual concentration distribution, the approach should at least shed some light on the presence or otherwise of putative species in the completely general description of solute self-association (Eq. (42a, b)), and hence on the reaction stoichiometry of protein self-association for the particular system under investigation.

### Analysis of two-state self-association involving tetramer

**Fig. 9:**
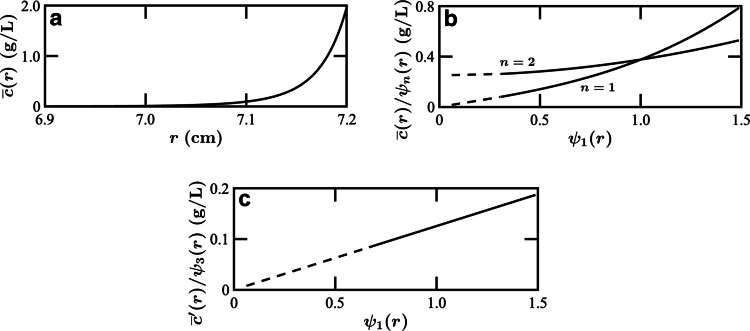
Delineation of the reaction stoichiometry for protein self-association from a simulated sedimentation equilibrium distribution for a dimer − tetramer system (${M}_{2}$ = 32,250 Da, $K_{2,4}$= 2.0 L/g). **a** The simulated concentration distribution at 30,000 rpm and 293 K. **b** Analysis of the distribution by means of Eq. (43) to establish the absence of monomer through a zero ordinate intercept for the dependence of $\overline c(r)/\psi_1(r)$ upon ${\psi }_{1}(r)$; and its reanalysis to estimate the dimer concentration at the reference radial position [${c}_{2}\left({r}_{F}\right)]$ as the ordinate intercept of the corresponding dependence (*n* = 2) with dimer the smallest species. **c** Analysis of the residual concentration distribution after subtraction of ${c}_{2}(r)$ to establish the absence of trimer and hence determine ${c}_{4}({r}_{F})$ as the slope of the linear dependence of $\overline{c }^{\prime}(r)/{\psi }_{3}(r)$ upon ${\psi }_{i}\left(r\right)$ are based on ${\psi }_{i}\left(r\right)$= 7.15 cm

Although the ultimate goal of the present investigation is to demonstrate a simple way of ascertaining the stoichiometry for more complicated self-association behaviour, it is helpful to illustrate the potential of the approach first with its application to a simple two-state system. For that purpose, we have simulated the concentration distribution in a sedimentation equilibrium experiment of meniscus-depletion design (Yphantis 1964) for a dimer-tetramer system with parameters based on those for methemoglobin at pH 6.0 (${M}_{2}$ = 32,250 Da, ${\overline v}\rho{_s}$= 0.755, and an apparent dimerization constant ${X}_{\mathrm{2,4}}^{app}$ of 2.0 L/g (Hensley et al. 1975; Jacobsen and Winzor 1995)) in which the total concentration ranged from essentially zero to 2.00 g/L in a liquid column with 6.900 and 7.200 cm as its radial extremities. Separate sedimentation equilibrium distributions for dimer and tetramer were calculated (Eq. (3) with $c_i(r)$ replacing ${z}_{i}(r)/{\gamma }_{i}(r)$) on the basis of respective concentrations of 0.78 and 1.22 g/L at the cell base, a rotor speed of 30,000 rpm, and *T* taken as 20 °C; and then combined to give the radial dependence of $\overline c(r)$ shown in Fig. 9a.

Inasmuch as the simulated concentration dependence presented in Fig. 9a only takes into account a simple dimer − tetramer equilibrium, the goal of the present approach is to establish the absence of monomeric and trimeric entities. To that end, Fig. 9b summarizes the application of Eq. (43) to test for the presence of monomer in the simulated distribution in Fig. 9a for the dimer-tetramer system, the solid line again being terminated at the value of ${\psi }_{1}\left(r\right)$ for which $\overline{c }\left(r\right)=0.025$ g/L. As required, the dependence of $\overline{c }(r)/{\psi }_{1}(r)$ upon ${\psi }_{1}\left(r\right)$ signifies an ordinate intercept of zero, and hence of an estimate of zero for ${c}_{1}({r}_{F})$, the concentration of monomer at the selected reference radial position (7.15 cm).

The demonstrated absence of monomer justifies reanalysis of the $\overline{c }\left(r\right)\,vs\,r$ distribution with dimer now the smallest species because the residual total concentration in Eqs. (44) and (45) remains the initial total concentration. As required, the finite ordinate intercept of that plot (second curve in Fig. 9b) of the dependence of $\overline{c }(r)/{\psi }_{2}(r)$ upon ${\psi }_{1}\left(r\right)$ identifies not only the presence of dimer but also its concentration, ${z}_{2}\left({r}_{F}\right) \approx {c}_{2}({r}_{F})$, at the reference radial distance (7.15 cm). Calculation of dimer distribution as ${c}_{2}\left(r\right)={c}_{2}({r}_{F}){\psi }_{2}(r)$ then allows delineation of the next residual concentration distribution, $\overline {c }^{\prime}(r)$ *vs* *r*, in which trimer is the smallest putative species. As well as confirming a value of zero for ${c}_{3}({r}_{F})$, the resulting dependence of $\overline{c }^{\prime}(r)/{\psi }_{3}(r)$ upon ${\psi }_{1}\left(r\right)$ is linear (Fig. 9c), and therefore signifies that the residual distribution is that for tetramer. As required, the slope of that line duplicates the value of ${c}_{4}({r}_{F})$ at the reference radial distance.

By eliminating the presence of monomeric and trimeric species in the simulated distributions, the present approach has achieved its goal of identifying the stoichiometry of self-association as a dimer-tetramer equilibrium. In an experimental context, a global estimate of *X*_24_ from several sedimentation equilibrium distributions would then be obtained most simply from nonlinear least-squares curve-fitting to the expression

46 $$\overline{c }\left(r\right)={{c}_{2}({r}_{F})\psi }_{2}\left(r\right)+{X}_{24}^{app}[{c}_{2}({r}_{F}){\psi }_{2}(r){]}^{2}$$

with the reference radial position in each distribution selected on the basis of a common $\overline{c }\left(r\right).$ On the grounds that ${X}_{2}$ and ${c}_{2}({r}_{F})$ now become the only parameters to be avaluated by nonlinear regression analysis, the precision of their estimates should be enhanced considerably by prior establishment of the self-association stoichiometry.

### Possibility of distinction between models of insulin self-association

From the first study of insulin self-association by direct analysis of sedimentation equilibrium distributions (Jeffrey et al. 1976), it was found by Omega analysis that the dependence of monomer concentration upon total protein concentration was best described in terms of monomer dimerization followed by isodesmic indefinite self-association of dimer, a minimalist model with monomer and even-numbered oligomers (dimer, tetramer, etc.) as the species present in equilibrium coexistence. Subsequent identification of two potential association sites in the crystal structure of the insulin monomer (Wollmer et al. 1980) prompted reanalysis of the same results in terms of indefinite self-association involving two separate isodesmic constants for head-to-head and tail-to-tail interaction between the two monomer sites (α and β) to form an equilibrium mixture comprising monomer and all (odd-numbered as well as even-numbered) oligomeric species (Nichol et al. 1984; Mark et al. 1987).

For the earlier model entailing a monomer − dimer equilibrium followed by isodesmic indefinite association of dimer (Jeffrey et al. 1976), the total weight concentration $\left(\overline{c }\right)$ and the weight concentration of monomer $({c}_{1})$ are related by the expression (assuming thermodynamic ideality)

47 $$\overline{c }={c}_{1}\left[1 +\frac{2{(K}_{2}/{M}_{1}){c}_{1}}{[1-{(K}_{2}/{M}_{1})({K}_{i2}/{M}_{1}){c}_{1}^{2}{]}^{2}}\right]$$

where $K_2$ is the molar association constant for dimerization and $K_{i2}$ is the molar isodesmic constant for the indefinite association of dimer.

The corresponding expression for the subsequent model (Nichol et al. 1984; Mark et al. 1987) refers to indefinite self-association of a bivalent monomer with nonidentical association sites (α and β) via α − α and β − β interactions. Linear chain growth then proceeds by successive addition of monomer to generate odd-numbered polymers with an α-site at one end and a β-site at the other, and even-numbered polymers terminated by either two α-sites or two β-sites. For this system with two isodesmic association constants (${K}_{i\alpha }$ and ${K}_{i\beta }$), the corresponding relationship between total weight-concentration of protein and its monomer counterpart becomes (Nichol et al. 1984; Mark et al. 1987)

48 $$\overline{c }={c}_{1}\left[\frac{[1+2({K}_{i\alpha }/{M}_{1}){c}_{1}][1+2({K}_{i\beta }/{M}_{1}){c}_{1}]}{[1-4({K}_{i\alpha }/{M}_{1})({K}_{i\beta }/{M}_{1}){c}_{1}^{2}]}\right]$$

which again allows simulation of the total weight-concentration distribution for a predetermined counterpart in terms of monomer concentration.

**Fig. 10:**
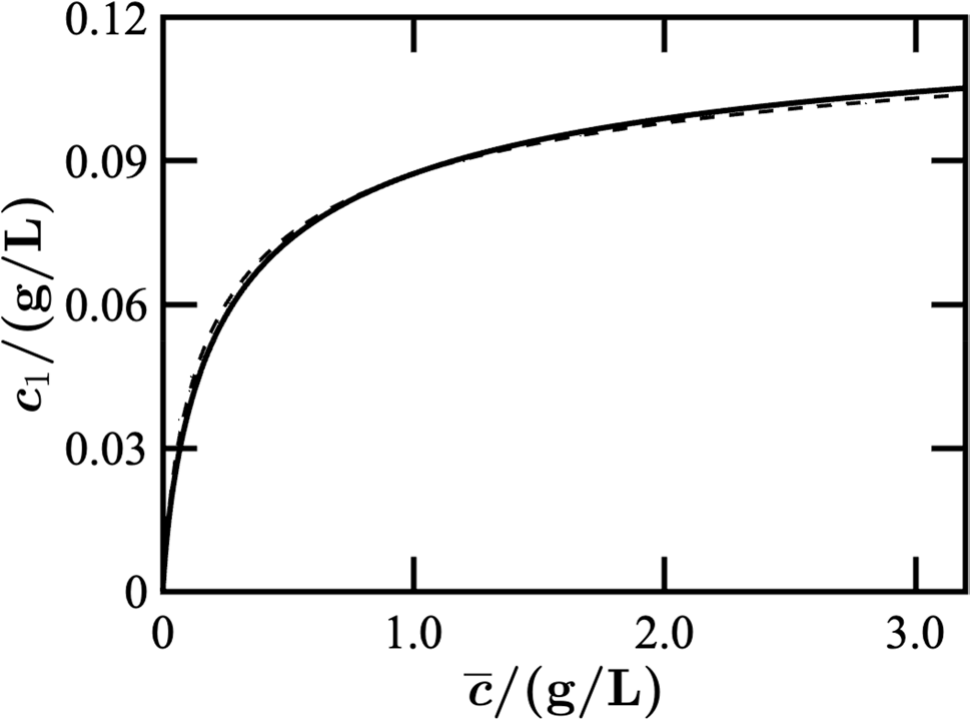
Self-association of zinc-free insulin at pH 7.0 (Jeffrey et al. 1976). Solid line (**——**), concentration dependence of monomer concentration calculated from Eq. (47) for a system involving isodesmic association of dimer $({K}_{2}/{M}_{1} =$ 19.2 L/g, ${K}_{i,2}/{M}_{1}=2.96$ L/g, Jeffrey et al. 1976). Dashed line (^**---**^), corresponding dependence calculated from Eq. (48) for a system involving two isodesmic associations of a bivalent monomer (${K}_{i\alpha }/{M}_{1}$ = 10.0 L/g; ${K}_{i\beta }/{M}_{1}$ = 1.48 g/L, Nichol et al. 1984)

In the initial analytical approach to the characterization of insulin self-association by sedimentation equilibrium (Jeffrey et al. 1976), delineation of the most appropriate model was based on the best-fit descriptions of the dependence of $\overline{c }(r)$ upon ${z}_{1}(r)$, expressed as $\overline z(r_F)\psi_1(r)$, for a range of models. However, as noted subsequently (Nichol et al. 1984), experimentally indistinguishable dependencies emanate from Eqs. (47) and (48). That finding is reinforced by Fig. 10, which compares the original best-fit dependence (^**_____**^) based on insulin dimerization followed by isodesmic self-association of dimer with $K_2/M_1=19.2$L/g and $K_{i2}/M_1$= 2.96 L/g (Jeffrey et al. 1976) with that (**– – –**) based on two different isodesmic self-associations ($K_{i\alpha}/M_1$=10.0 L/g ${K}_{i\beta }/{M}_{1}$ = 1.48 L/g) of a bivalent monomer (Nichol et al. 1984). Establishing the existence of odd-numbered oligomers is required to justify elimination of the earlier model as the simplest thermodynamic description of insulin self-association at neutral pH.

The point at issue is whether the present approach to delineating reaction stoichiometry has potential to identify the presence of trimer and hence to provide additional support for the later model of insulin self-association (Nichol et al. 1984; Mark et al. 1987). For systems involving indefinite self-association, the problems of employing the statistical-mechanical approach to allowance for thermodynamic nonideality become prohibitive even at the level of the second virial coefficient. We have therefore again simulated sedimentation equilibrium distributions that cover a sufficiently small concentration range to justify the substitution of solute concentrations ${c}_{i}\left(r\right)$ for their thermodynamic activity counterparts ${z}_{i}\left(r\right).$

### Analysis of a distribution for isodesmic association of dimer

**Fig. 11:**
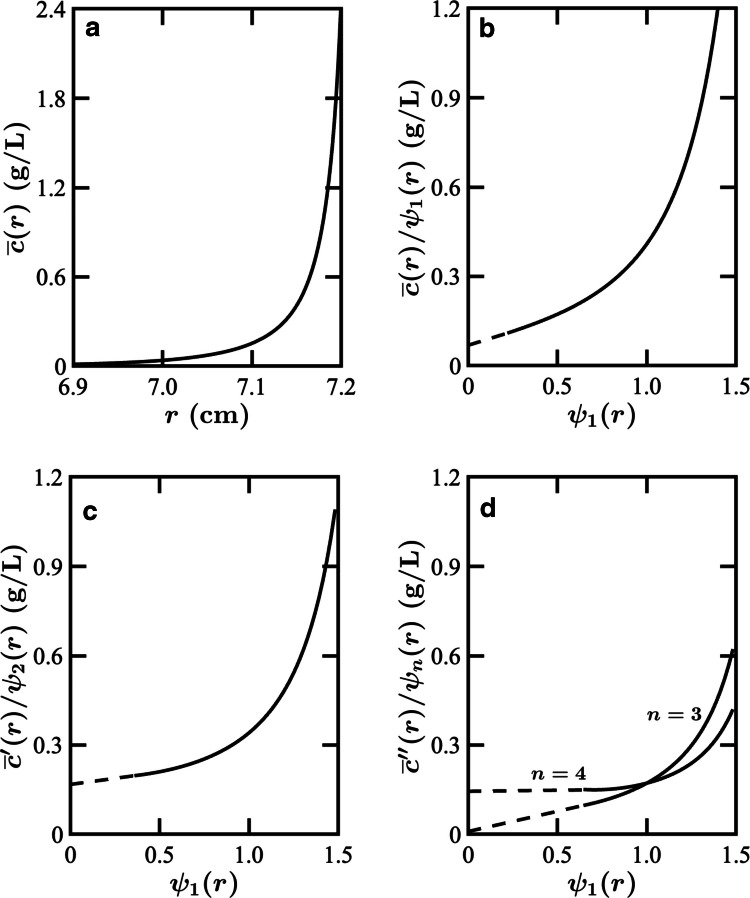
Identification of oligomer species involved from a simulated sedimentation distribution for the model of insulin self-association involving isodesmic self-association of dimer. **a** The simulated concentration distribution at 40,000 rpm and 25 °C for the reported system (Jeffrey et al. 1976) with ${K}_{2}$ = 110,000 M^−1^ and ${K}_{i2}$ = 17,000 M^−1^. **b** Psi analysis of the $\overline{c }(r)$ distribution to obtain the monomer concentration ${c}_{1}\left({r}_{F}\right)$ at the reference radial position (${r}_{F}$) as the ordinate intercept for the distribution with 6.90 ≤ *r* ≤ 7.20 cm. **c** Evaluation of the reference dimer concentration, ${c}_{2}({r}_{F})$, from the corresponding Psi analysis of the residual concentration distribution, $\overline{c }^{\prime}(r)$ = $\overline{c }\left(r\right)-{c}_{1}(r)$.** d** Demonstrated absence of trimer from Psi analysis (with *n* = 3) of the subsequent residual total concentration distribution $\overline{c }^{\prime \prime}(r)=\overline{c }^{\prime}(r)-{c}_{2}(r)$, and evaluation of ${c}_{4}\left({r}_{F}\right)$ from Psi analysis (with *n* = 4) of the same distribution

A sedimentation equilibrium distribution for insulin consonant with Eq. (47) has been obtained by using Eq. (1) with $$i=1$$ to calculate the distribution of an ideal monomer ($${M}_{1}=5734$$ Da, $${\overline v}\rho{_s}$$ = 0.73mL/g) and a liquid column with radial extremities (*r*_*a*_*, r*_*b*_) of 6.90 and 7.20 cm subjected to centrifugation at angular velocity corresponding to 40,000 rpm, a temperature of 25 °C and *r*_*F*_ = *r*_*b*_ = 7.20 cm. The reported values of 11 × 10^4^ M^−1^ and 1.7 × 10^4^ M^−1^ for $$K_2$$ and $${K}_{i2}$$, respectively (Jeffrey et al. 1976), were used to generate the ideal monomer distribution by setting $${c}_{1}({r}_{F})$$ = 0.107 g/L. This then yielded the complete sedimentation equilibrium distribution [$$\overline{c }\left(r\right)\,vs$$* r*] shown in Fig. 11a. In that regard, it is worth noting that the small size of insulin monomer (5734 Da) precludes the attainment of an equilibrium distribution with a protein concentration of essentially zero in the meniscus region. Because $$\overline{c }\left({r}_{a}\right)=0.13$$ g/L (or 0.43 fringe) in Fig. 11a, its current experimental delineation would have required the combination of a synthetic boundary run (to define $${J}_{o})$$ with identification of the hinge-point from concurrent absorbance distributions at the beginning and end of the run (see above).

For Psi analysis of the distribution in Fig. 11a (Eq. 43)), we chose a more appropriate reference radial position (${r}_{F}$) of 7.15 cm. Figure 11b shows the dependence of $\overline{c }(r)/{\psi }_{1}(r)$ upon ${\psi }_{1}(r)$, the limiting slope (**——**) of which yields an estimate of 0.068 g/L for the ordinate intercept, ${c}_{1}({r}_{F})$. This value agrees well with the theoretical value of 0.069 g/L for the input $\overline{c }({r}_{F})$ of 0.409 g/L. Termination of the solid line in Fig. 11b again reflects a lower-limiting value of 0.025 g/L (0.08 fringe) for $\overline{c }\left(r\right).$ In the present context, the point at issue is whether the suggested procedure for determining the concentrations of dimer and higher oligomers from successively depleted residual total concentration distributions can supply sufficient information to identify the mode of self-association.

Subtraction of ${c}_{1}\left(r\right)={c}_{1}\left({r}_{F}\right){\psi }_{1}(r)$ from $\overline{c }(r)$ throughout the simulated sedimentation equilibrium distribution gives rise to a residual total concentration distribution $\overline{c }^{\prime}(r)$ as a function of $r$, in which dimer is now the smallest putative species. Analysis of that residual concentration distribution in terms of Eq. (45) with ${r}_{F}$ maintained at 7.15 cm is shown in Fig. 11c, where the best-fit limiting slope (− − −) of the dependence of $\overline c^{\prime}\left(r\right)\psi_2\left(r\right)$ upon ${\psi }_{1}(r)$ signifies an ordinate intercept, $c_2(r_F)$, of 0.168 g/L. In view of the length of extrapolation required to accommodate the need for truncation of the data set (**——**) at $\overline{c }^{\prime}(r)=0.025$ g/L, this value of ${c}_{2}({r}_{F})$ correlates reasonably well with its theoretical counterpart [$2{K}_{2}{c}_{1}({r}_{F}{)}^{2}$] of 0.174 g/L. Indeed, the result from Fig. 11c encourages one to continue this approach by calculating $\overline{c }^{\prime \prime}(r)$ =$\overline{c }^{\prime}(r)- {c}_{2}\left({r}_{F}\right){\psi }_{2}\left(r\right).$

Reward for perseverance with this approach is evident in Fig. 11d (*n* = 3), where the dependence of $\overline{c }^{\prime \prime}(r)/{\psi }_{3}(r)$ upon ${\psi }_{1}(r)$ has a limiting best-fit slope commensurate with an ordinate intercept of 0.01 g/L. In other words, the analysis has confirmed the absence of trimer in the self-association pattern. Additional support for the particular model of indefinite self-association is provided by analysis of the same residual concentration distribution to establish the presence of tetramer in Fig. 11d (*n* = 4), where the ordinate intercept, ${c}_{4}({r}_{F})$, of the dependence of $\overline{c }^{\prime \prime}(r)/{\psi }_{4}(r)$ upon ${\psi }_{1}(r)$ is finite (0.145 *cf* 0.090 g/L). Furthermore, the essentially zero slope of the extrapolation in Fig. 11d signifies an absence of free pentamer at the reference radial position, ${c}_{5}({r}_{F})\approx 0$.

It therefore appears that resort to the Psi analysis of two residual total concentration distributions [$\overline{c }^{\prime}\left(r\right)$ and $\overline{c }^{\prime \prime}(r)$] would have sufficed to provide model-independent substantiation of the scheme involving isodesmic association of dimeric insulin (Jeffrey et al. 1976). The next step involves application of the same approach to the corresponding simulated distribution for the alternative model of insulin self-association (Nichol et al. 1984; Mark et al. 1987) to determine whether distinction between the two models can be achieved by this procedure.

### Analysis of a distribution for isodesmic associations of a bivalent monomer

**Fig. 12:**
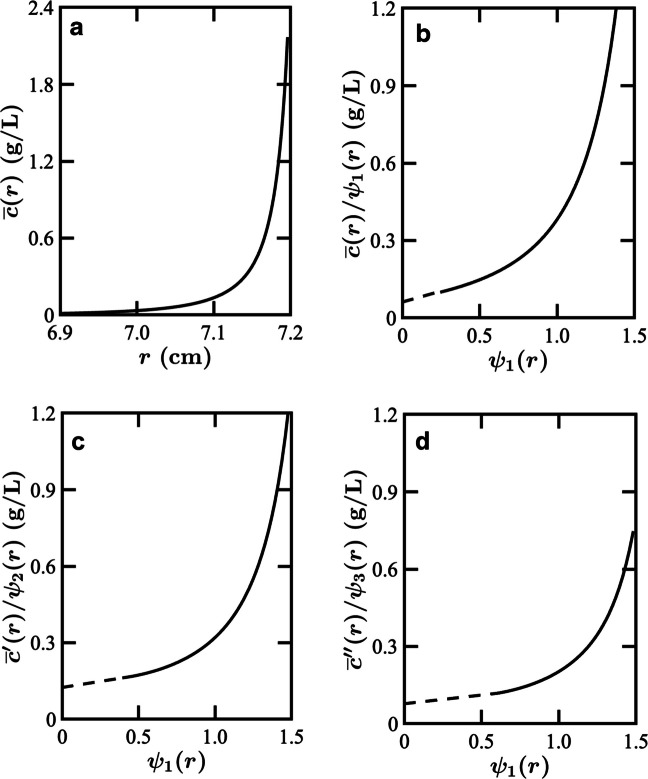
Identification of oligomeric species involved from a simulated distribution (40,000 rpm, 20 °C) for the model of insulin self-association involving two isodesmic associations of a bivalent monomer. **a** The simulated concentration distribution generated by Eq. (48) with ${K}_{i\alpha }=\mathrm{57,500}$ M^−1^, ${K}_{i\beta }$ = 8,500 M^−1^ and the monomer distribution used in Fig. 11. **b** Psi analysis to determine ${c}_{1}\left({r}_{F}\right)$ as the ordinate intercept. **c** Corresponding Psi analysis of the residual total concentration distribution $\overline{c }^{\prime}(r)$ to obtain ${c}_{2}({r}_{F})$. **d** Demonstrated presence of trimer by Psi analysis of the $\overline{c }^{\prime \prime}(r)$ distribution

The simulated sedimentation equilibrium distribution for this model (Fig. 12a) has been generated on the basis of the ideal monomer distribution used in Fig. 11a and the $\overline{c }\left(r\right)\;vs$ *r* counterpart obtained from Eq. (48) with respective values of 10.0 and 1.48 g/L for ${K}_{i\alpha }/{M}_{1}$ and ${K}_{i\beta }/{M}_{1}$, as in Fig. 10. Analysis of those results in terms of Eq. (43) with the same reference radial position (7.15 cm) gives rise to the dependence of $\overline{c }(r)/{\psi }_{1}\left(r\right)$ upon ${\psi }_{1}(r)$ presented in Fig. 12b and an ordinate intercept of 0.062 g/L. This estimate of ${c}_{1}({r}_{F})$ would again be indistinguishable experimentally from the input value of 0.069 g/L for $\overline c(r_F)$ = 0.383 g/L. Subtraction of ${c}_{1}(r)$ from $\overline{c }(r)$ throughout the distribution then generated its residual for total concentration counterpart, ${\overline{c} }^{\prime}(r)$, which was analyzed (Fig. 12c) via Eq. (45) to obtain an ordinate intercept, ${c}_{2}\left({r}_{F}\right),$ of 0.125 g/L from the dependence of $\overline{c }^{\prime}(r)$/${\psi }_{2}(r)$ upon ${\psi }_{1}(r)$. This slightly high estimate of the theoretical value, $2\lbrack(K_{i\alpha}+K_{i\beta})/M_1\rbrack{(c_1r_F)}^2$= 0.110 g/L, presumably reflects the immediately previous underestimation of ${c}_{1}\left({r}_{F}\right)$, and hence the corresponding overestimation of the ${\overline{c} }^{\prime}(r)$ distribution.

Continuation of the analysis by subtracting ${c}_{2}(r)$ from $\overline{c }^{\prime}(r)$ to obtain the second residual total concentration distribution $\overline{c }^{\prime \prime}(r)$ with trimer the smallest species is summarized in Fig. 12d, which presents the dependence of $\overline{c }^{\prime \prime}(r)/{\psi }_{3}(r)$ upon ${\psi }_{1}(r)$. The important outcome from this dependence is the observation of a finite ordinate intercept, ${c}_{3}({r}_{F})$, of 0.078 g/L. In that regard, the disparity between the estimate and its theoretical counterpart, $12({K}_{i\alpha }/{M}_{1})({K}_{i\beta }/{M}_{1}){c}_{1}^{3}$ = 0.059 g/L, can be attributed to the increasing uncertainty due to systematic error in each successive residual total concentration distribution. Despite that quantitative limitation, this stepwise approach has provided unequivocally a means of detecting the presence of trimer in the system giving rise to the sedimentation equilibrium distribution. Presence of an odd-numbered oligomeric series would be established by following this procedure.

The demonstrated ability to identify the different reaction stoichiometries from the two simulated distributions (Figs. 11 and 12) despite their return of indistinguishable ${c}_{1}\left(r\right)\,vs\,\overline{c }(r)$ dependencies (Eqs. (47), (48) and Fig. 10) establishes the potential of the analytical approach to experimental delineation of the stoichiometry of protein self-association in complicated situations.

## Discussion

This review has outlined research over the past five decades into the development of improved procedures for the quantitative characterization of protein self-association by direct analysis of sedimentation equilibrium distributions. That sets it apart from most current sedimentation equilibrium studies of protein self-association, where the emphasis has been directed towards the incorporation of advances in computer technology that have allowed the analytical approaches of the 1950 s and 1960 s to be replaced by the highly iterative and model-dependent numerical simulation procedures in programs such as NONLIN (Johnson et al. 1981), SEDANAL (Sherwood and Stafford 2016a, b), SEDFIT (Schuck 2016), and SEDNTERP (Philo 2023). For thermodynamically ideal systems, both the analytical (this work) and numerical simulation procedures suffice for characterizing two-state (or possibly three-state) self-association because of their reliance upon the same quantitative expression (Eq. (4a) or (4b)). However, the analytical approach provides direct and model-independent access to $${c}_{1}({r}_{F})$$, the monomer concentration at reference radial distance *r*_*F*_, from the dependence of $$\overline{c }(r)/{\psi }_{1}(r)$$ upon $${\psi }_{1}(r)$$ (Eq. (43)), and hence to $${c}_{1}(r)$$ throughout the [$$r, \overline{c }(r)$$] distribution via Eq. (3). The only model dependence is thus encountered in the evaluation of best-fit estimates for $${K}_{2},$$$${K}_{3}$$, etc. that emanate from nonlinear least-squares analysis of the transformed data set, $$\overline{c }\left(r\right)\,vs\,{c}_{1}\left(r\right),$$ in terms of Eq. (4a) with a value of unity for all activity coefficients $${\gamma }_{i}(r)$$. Indeed, there is even potential for removing that model dependence by prior identification of the oligomeric species involved in the self-association (Figs. 9, 11, and 12).

A major reason for persisting with the analytical approach to characterization of protein self-association was the potential for accommodating the consequences of thermodynamic nonideality because of its ability to cope iteratively with the composition dependence of activity coefficients that arise in excluded volume considerations of the problem (McMillan and Mayer 1945; Hill 1959; Wills et al. 1980). However, progress in that endeavour had to await delineation of the concentration scale and corresponding thermodynamic activity most appropriate for analysis of sedimentation equilibrium experiments. That identification of the thermodynamic activity defined at constant solvent chemical potential, µ_*s*_, (Wills and Winzor 1992; Wills et al. 1993) paved the way for the direct application of McMillan − Mayer theory, and thereby ended the indecision about whether the experimentally measured second virial coefficient is ${B}_{22}$ (Ogston and Winzor 1975; Minton 1983) or ${B}_{22}+{M}_{2}{\overline{v} }_{2}$ (Ross and Minton 1977; Wills et al. 1980). The ${M}_{2}{\overline{v} }_{2}$ molar volume term only comes into play when an experimenter is using the molar (or g/L) scale to monitor a molal thermodynamic activity—the situation pertaining, for example, in static light scattering studies (Winzor et al. 2007b; Wills et al. 2015).

Potential difficulties associated with the introduction of additional iteration into the earliest numerical simulation program (NONLIN, ORIGIN) may well have prompted the decision (Johnson et al. 1981) to incorporate allowance for the consequences of thermodynamic nonideality on the basis of a physically unrealistic assumption (Adams and Fujita 1963) about the inter-relationship between activity coefficients (Eq. (5)) for self-associating species. As noted in the discussion of Eq. (5), it seems incredible that the procedure reigned supreme for three decades as a means of refining values of $K_i$ (or $X_i$) despite its prediction that thermodynamic nonideality should have no effect on the magnitude of the association constant (Jacobsen and Winzor 1992). Meanwhile, Hill and Chen (1973) had already provided a means for retaining the expression of activity coefficients in terms of total protein concentration $\overline{c }(r)$ that accommodated the composition dependence of ${\gamma }_{i}$(*r*) emanating from their consideration on the statistical-mechanical basis of excluded volume (McMillan and Mayer 1945; Hill 1959). Our decision to explore this previously neglected means of avoiding the adoption of iterative procedures in the analytical approach (Wills and Winzor 1992) would obviously also have implications for the numerical simulation procedures.

In the only experimental application of the Hill-Chen approach to sedimentation equilibrium distributions (Wills and Winzor 1992), the earlier results for lysozyme (Wills et al. 1980) were analyzed according to the logarithmic form of Eq. (22), which necessarily converges slowly because of its origin (Eq. (21)) as a series expansion in terms of ${C}_{1}(r)$. The fact that polynomial curve-fitting of that dependence of $\mathrm{ln}[{z}_{1}\left(r\right)/\overline{C }\left(r\right)]$ upon $\overline{C }(r)$, Fig. 2a, only yielded acceptable estimates of the linear and quadratic coefficients prompted the development of a modified Hill-Chen approach (Wills et al. 1996) in which $\overline{C }(r)$ was expressed (Eq. (26)) as a polynomial series in ${z}_{1}\left(r\right)={z}_{1}\left({r}_{F}\right){\psi }_{1}(r)$, where $z_i(r_F)$ is a constant (the monomer thermodynamic activity at the selected reference radial position). Although polynomial curve-fitting of the dependence of $\overline{C }(r)$ upon ${\psi }_{1}(r)$ for the same lysozyme data (Fig. 2b) yielded estimates of ${z}_{1}\left({r}_{F}\right)$, ${K}_{2}$, and ${K}_{3}$ with seemingly reasonable precision, an inability to obtain meaningful polynomial coefficients beyond the quadratic term in ${\psi }_{1}(r)$ was again a cause for concern about the reliability of the equilibrium constant estimates. Attention was therefore switched to more detailed studies of α-chymotrypsin at pH 4, a simpler system for which self-association is restricted to reversible dimerization.

Similar problems were encountered in initial applications (Wills et al. 1996) of the modified Hill-Chen (or Psi) procedure to studies of α-chymotrypsin dimerization (Fig. 4) because of the necessity to truncate the polynomial curve-fitting at the quadratic term in Eq. (24). Of particular note is the estimate of 3.0 × 10^4^ M^−1^ deduced from Fig. 4b, which is considerably smaller than that of 4.6 × 10^4^ M^−1^ obtained (Fig. 3) by subjecting the same [$r, \overline{C }(r)$] data set (pH 3.9, *I*_*M*_ 0.2) to iterative analysis with activity coefficients based on the consequences of nearest-neighbor interactions (Eqs. (31a, b)).

The necessity for experimental delineation of magnitudes for higher virial coefficients in Eq. (24) is established unequivocally by analyses of a high-speed sedimentation distribution (Fig. 5) obtained for α-chymotrypsin (pH 4.1, *I*_*M*_ 0.05) in a Beckman XL-I instrument. Truncation of the polynomial curve-fit of the dependence of $\overline{C }(r)$ upon ${\psi }_{1}\left(r\right)$ at the quadratic term clearly leads to a lower estimate of ${K}_{2}$ than that deduced by including the [${\psi }_{1}\left(r\right){]}^{3}$ term in the curve-fit (Fig. 6b). Its extension to the quartic term leads to relatively small change in the ${K}_{2}$ estimate. From Fig. 6b, it is also evident that essentially the same dimerization constant emanates from iterative analysis with activity coefficients based on nearest-neighbour interactions (Eqs. (31a, b))—a value that is little changed by using more complicated expressions (Eq. 34) for the activity coefficients.

The above findings for α-chymotrypsin have highlighted a limitation of the modified Hill-Chen approach to quantifying nonideal protein self-association on the basis of its thermodynamic description as a single-solute system because of the necessity for meaningful experimental delineation of the polynomial in ${\psi }_{1}(r)$, Eq. (24), beyond the quadratic term in order to obtain a reliable estimate of ${K}_{2}$. Its application to systems involving oligomeric states larger than dimer would therefore require the experimental delineation of polynomial coefficients in Eq. (24) extended to terms in $[{\psi }_{1}(r){]}^{5}$ and higher, a requirement not achievable with the current Rayleigh interference optical system incorporated into the Beckman XL-I ultracentrifuge. There would be little option other than to revert to the more cumbersome but manageable iterative approach in which the magnitudes of self-association constants are refined by means of composition-dependent activity coefficients based on nearest-neighbor interactions.

Although the association constant can be determined with reasonable precision, its accuracy depends upon the assigned magnitudes of monomer radius ${R}_{1}$ and net charge ${Z}_{1}$ that are incorporated into calculations of the second virial coefficients ${B}_{11}^{*}$ and ${B}_{1n}^{*}$ (Eqs. (8a) and (11)). In that regard, the substitution of the experimental Stokes radius for ${R}_{1}$ should suffice, as should the approximations that ${R}_{n}={n}^{1/3}{R}_{1}$ and ${Z}_{n}=n{Z}_{1}$. The quantity of greatest concern is the value to be assigned to the monomer valence ${Z}_{1}$, which clearly has a major impact on the estimated magnitude of the association constant (Fig. 7a). Because protein valence is a parameter that is rarely measured, the value deduced from the amino acid composition is probably the next best option. Fine tuning of the monomer valence can then be attempted on the basis of the goodness-of-fit of the values of ${Z}_{1}$ on either side of amino acid composition value (as in Fig. 7a). This is the only option when scaled-particle theory (Chatelier and Minton 1987) is used for the determination of activity coefficients (Fig. 7b) because the consequences of net charge are accommodated empirically by increasing the effective radius of an uncharged spherical monomer (Minton and Edelhoch 1983).

Considerations thus far have revealed the difficulties/uncertainties inherent in the quantitative characterization of nonideal protein self-association in instances where the reaction stoichiometry is already known. That situation worsens when the stoichiometry is also being sought from the same sedimentation equilibrium distributions. The approach would obviously benefit from experimental delineation of the self-association pattern before resort is made to nonlinear least-squares curve-fitting. In that regard, there are clearly limitations to the suggested procedure for identifying the presence of successive oligomers from the dependence of $\overline c^\dagger(r)/\psi_n(r)$ upon ${\psi }_{1}(r)$, where $\overline c^\dagger(r)$ is a residual total concentration [${\overline{c} }^{\prime}\left(r\right), \overline{c }^{\prime \prime}(r)\;\mathrm{etc}.]$ after subtracting the free concentrations of already identified oligomeric states (Eqs. (43) and (45)). However, application of this procedure to simulated sedimentation equilibrium data signifies the feasibility of employing the approach as a way of identifying a limited self-association equilibrium such as dimer-tetramer (Fig. 9). Furthermore, such treatment of simulated data for insulin (Figs. 10 − 12) offers promise for the possibility of distinguishing between various modes of isodesmic indefinite protein self-association (Van Holde and Rossetti 1967; Pekar and Frank 1972; Adams et al. 1975; Nichol et al. 1984) from a few iterations of the residual concentration analysis. This aspect of the analytical approach certainly warrants further experimental investigation.

In summary, the goal of our research has been to take advantage of the scientific breakthrough by Goldberg (1953) with the demonstration that the technique of sedimentation equilibrium could be accorded full thermodynamic status by expressing the opposing force to sedimentation as $(\partial {\mu }_{2}/\partial {c}_{2}{)}_{r}$ rather than $D(\partial {c}_{2}/\partial r{)}_{r}$, the term used by Lamm (1928) in the continuity equation describing solute migration in a centrifugal field. That breakthrough has allowed the consequences of thermodynamic nonideality to be incorporated into the analysis of sedimentation equilibrium distributions on the statistical-mechanical basis of excluded volume (McMillan and Mayer 1945; Hill 1959). The use of the analytical approach for this purpose has demonstrated the need for changes in the following technical as well as theoretical aspects of the more popular numerical simulation approaches, which rely solely on curve-fitting for the evaluation of parameters:

(i) Analysis should be restricted to sedimentation equilibrium distributions of meniscus depletion design to allow experimental delineation of the baseline and hence remove uncertainty from the definition of $\overline{c }(r)$ throughout the distribution. Alternatively, the current practice of employing sedimentation equilibrium distributions at a range of rotor speeds could be retained by locating the hinge point (where $J\left(r\right)={J}_{o}$) from the intersection point of absorbance records of the initial and final distributions.
(ii) Modification of the numerical simulation programs to deduce ${z}_{1}({r}_{F})$ and hence ${z}_{1}\left(r\right)$ as a function of $\overline{C }(r)$ throughout a sedimentation equilibrium distribution would have the advantage of decreasing the number of quantities to be deduced as curve-fitting parameters, as would prior experimental identification of oligomers involved in the self-association process.
(iii) Additional iteration will still need to be introduced into the analysis to accommodate the composition dependence of activity coefficients.
(iv) Evaluation of an association equilibrium constant ${K}_{n}$ from those resultant curve-fitting parameters is dependent upon the assignment of magnitudes to the three second virial coefficients (${B}_{11}^{*},{B}_{1n}^{*},\mathrm{and}\,{B}_{nn}^{*}$) that appear in the dependence of $\overline{c }(r)$ upon ${\psi }_{1}(r)$ for situations where description of nonideality in terms of nearest-neighbor interactions suffices.

The stage is now set for these scientific developments in sedimentation equilibrium, gathered over the past five decades, to be incorporated into online programs that will afford the ultracentrifuge community access to more accurate quantification of protein self-association than is available from current counterparts.

## Data Availability

No datasets were generated or analysed during the current study.
