# Correction to: A quest for greater thermodynamic rigour in the quantitative characterization of protein self-association by direct assessment of sedimentation equilibrium distributions

**DOI:** 10.1007/s12551-026-01415-0

**Published:** 2026-05-25

**Authors:** Donald J. Winzor, Peter R. Wills

**Affiliations:** 1 School of Chemistry and Molecular Biosciences, University of Queensland, Brisbane, Queensland 4072 Australia; 2 Department of Physics, University of Auckland, Private Bag 92019, Auckland, New Zealand

**Correction to** **:** **Biophysical Reviews**

10.1007/s12551-025-01371-1

This erratum is to inform readers of typesetting errors that appeared in the published version of the above-referenced article. These errors do not affect the overall conclusions or scientific validity of the work, but we believe it is important to correct them for clarity and accuracy. We apologize for any confusion or inconvenience these errors may have caused.

The original article has been corrected.

